# Bridging the Transient Intraluminal Stroke Preclinical Model to Clinical Practice: From Improved Surgical Procedures to a Workflow of Functional Tests

**DOI:** 10.3389/fneur.2022.846735

**Published:** 2022-03-11

**Authors:** Raquel Pinto, Ana Magalhães, Mafalda Sousa, Lúcia Melo, Andrea Lobo, Pedro Barros, João R. Gomes

**Affiliations:** ^1^Molecular Neurobiology Unit, IBMC-Instituto de Biologia Molecular e Celular, Porto, Portugal; ^2^I3S–Instituto de Investigação e Inovação em Saúde, Universidade do Porto, Porto, Portugal; ^3^Addiction Biology Unit, IBMC-Instituto de Biologia Molecular e Celular, Porto, Portugal; ^4^Advanced Light Microscopy Unit, I3S–Instituto de Investigação e Inovação em Saúde, Universidade do Porto, Porto, Portugal; ^5^Neurology Department, Centro Hospitalar de Vila Nova de Gaia/Espinho, Vila Nova de Gaia, Portugal; ^6^Stroke Unit, Centro Hospitalar de Vila Nova de Gaia/Espinho, Vila Nova de Gaia, Portugal

**Keywords:** stroke, tMCAO, translation, behavior, mice, patients

## Abstract

Acute ischemic stroke (AIS) remains a leading cause of mortality, despite significant advances in therapy (endovascular thrombectomy). Failure in developing novel effective therapies is associated with unsuccessful translation from preclinical studies to clinical practice, associated to inconsistent and highly variable infarct areas and lack of relevant post-stroke functional evaluation in preclinical research. To outreach these limitations, we optimized the intraluminal transient middle cerebral occlusion, a widely used mouse stroke model, in two key parameters, selection of appropriate occlusion filaments and time of occlusion, which show a significant variation in the literature. We demonstrate that commercially available filaments with short coating length (1–2 mm), together with 45-min occlusion, results in a consistent affected brain region, similar to what is observed in most patients with AIS. Importantly, a dedicated post-stroke care protocol, based on clinical practice applied to patients who had stroke, resulted in lower mortality and improved mice welfare. Finally, a battery of tests covering relevant fine motor skills, sensory functions, and learning/memory behaviors revealed a significant effect of tMCAO brain infarction, which is parallel to patient symptomatology as measured by relevant clinical scales (NIH Stroke Scale, NIHSS and modified Rankin Scale, mRS). Thus, in order to enhance translation to clinical practice, future preclinical stroke research must consider the methodology described in this study, which includes improved reproducible surgical procedure, postoperative care, and the battery of functional tests. This will be a major step s closing the gap from bench to bedside, rendering the development of novel effective therapeutic approaches.

## Graphical Abstract

Improving translation of tMCAO mice model. Improvement of the surgical procedure, post-operative care and functional evaluation in the tMCAO mice, gives rise to a model with high parallel and translation into clinical reality. Induction of ischemia for 45-mins using smaller commercial filaments gives rise to uniform lesion volume that together with the improved post-operative care, decreases this model mortality. Furthermore, this allows for intensive long-term functional evaluation of mice. This way, clinical symptoms and ethology, as well as scales used to evaluate stroke, are mimicked in a mouse model. 
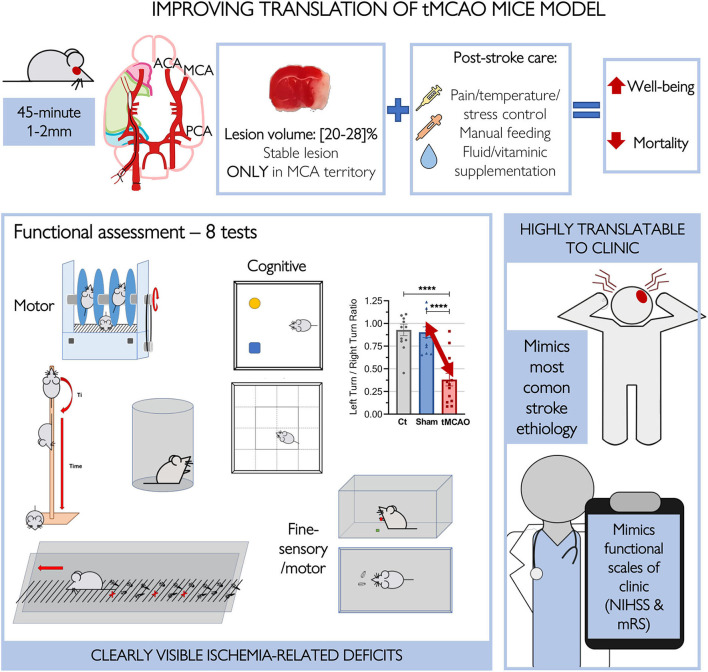

## Introduction

Ischemic stroke is a leading cause of death and disability worldwide (1). Currently, the only treatments available are focused on reperfusion *via* endovascular thrombectomy or intravenous thrombolysis. However, due to short-therapeutic windows, <15% of patients with acute ischemic stroke (AIS) are eligible for treatment (2). Many efforts have been employed to develop treatments that, when combined with thrombolytic techniques, can extend the interval for reperfusion for longer periods and/or ameliorate AIS symptoms. Several compounds have shown promising results in preclinical studies; however, they were unsuccessful in clinical trials, possibly associated to low predictive data and lack of robust preclinical evidence. Preclinical studies frequently sustain their findings on histological examination of post-stroke damage, but they miss more complex end points, such as long-term complete functional tests, to provide data in a “real-world” context (3, 4). In animal models of stroke, however, infarct size does not necessarily correlate with functional outcome, so this parameter is now considered insufficient to evaluate stroke lesions and the effectiveness of therapeutic strategies (5, 6). Conversely, the histological end point is in stark contrast with clinical end points, which use neurologic scores like the modified ranking scale (mRS) and National Institutes of Health Stroke Scale (NIHSS) to determine functional outcome and level of disability (7, 8). The mRS scale is usually the primary measure used in clinical trials to evaluate patient's recovery and functional dependence in their daily activities (1), while the NIHSS scale captures both motor and non-motor impairments and deficits over time (9). Considering that both patient and animal stroke models show different degrees of sensorimotor and memory impairments, researchers now agree that the assessment of behavior in animal models must complement the assessment of brain damage with histological techniques to produce more reliable and translatable results (10, 11).

Since mouse stroke behavioral phenotypes are highly heterogeneous and often affect several capabilities, a comprehensive functional assessment requires an extensive battery of tests to represent several specific aspects of functional behavior (12, 13). Several tests have been conducted to assess stroke severity, from measure of global neurological status, motor reflexes, and fine sensorimotor skills, to cognitive tests that assess learning/memory and anxiety/depression. However, no universal battery of behavioral tests exists in the field, and specific tests are applied and modified to meet the demands of particular studies.

The most prevalent cases of AIS in humans are caused by occlusion of the middle cerebral artery (MCA) and its branches (about 65%) (14–16), so the intraluminal suture of the MCA is the most commonly used animal model of ischemic stroke. This model induces cortical and subcortical infarction, is relatively non-invasive, allows for highly controllable reperfusion, leads to formation of a distinguishable penumbra, and, importantly, reproduces significant functional impairments observed in patients with stroke (1, 17). However, this model displays some pitfalls, such as inconsistency in the volume of ischemic lesions, which is a concern in animal models where consistent infarct size facilitates the analysis of intergroup variations both in functional and histological outcomes (18). Many variables are known to impact the resulting lesion and mortality in the intraluminal filament method of MCAO, constituting an ethical problem that impacts data robustness (19).

In this study, we improved relevant aspects of the model (filament coating length, occlusion time, and postoperative care), resulting in mice with consistent and less variable lesion sizes and lower mortality rates. Importantly, we have established a battery of behavioral tests to assess stroke severity, from fine sensorimotor skills to cognition. These will be crucial to improve translation from preclinical studies to clinical reality.

## Methods

### Animals

All experiments and procedures were approved by the Institutional and National General Veterinary Board Ethical Committees (approval reference number 003424), and are in line with National and European Union regulations. Four to six-month-old C57/BL6 mice of both sexes weighing 28.53 ± 0.74 g at the beginning of the experiment were used. The animals were maintained in type II cages under pathogen-free conditions (microbiological health status available) in an animal room with constant temperature (20–24°) and humidity (45–65 %), and in a reversed 12-h light/dark cycle (lights on at 12 am and lights off at 12 pm). The animals had access to regular rodent chow and tap water *ad libitum*. Randomization for group selection was performed using the GraphPad QuickCalcs web site: https://www.graphpad.com/quickcalcs/randomize1/. This study was not pre-registered. ARRIVE guidelines for reporting animal research were taken into consideration in the reporting of the experiments.

### Experimental Groups

The first cohort of animals was used to define the most appropriate tMCAO (time of occlusion + coating length combination) protocol. Three groups were assigned: tMCAO 45 min (*n* = 6), tMCAO 25 min (*n* = 5), and sham (*n* = 6); however, for mortality assessment, we included more animals that have been used in previous experiments [**Figure 2B**: tMCAO 45 min (*n* = 6), tMCAO 25 min (*n* = 12), and sham (*n* = 15)]. The animals in this cohort were treated with normal postoperative care. In the second cohort, three groups were also assigned: control (naïve mice) (*n* = 4–12), sham (*n* = 3–17), and tMCAO 45 min (*n* = 13–17). These animals were treated with optimized postoperative care and performed the designed functional workflow.

### Transient Middle Cerebral Artery Occlusion

Transient middle cerebral artery occlusion (tMCAO) was induced with an intraluminal suture using the Longa method (20–23). The mice were anesthetized with isoflurane (4% for induction and 2–1.25% for maintenance) in a mixture of N_2_O:O_2_ (70:30, using a small anesthesia system). Rectal temperature was maintained at 37.2°C throughout the surgical procedure using a rodent warmer with a rectal probe (Stoelting, Wood Dale, IL, United States). Regional cerebral blood flow (rCBF) within the MCA territory was monitored transcranially using a laser Doppler flowmeter (LDF; PeriFlux System, Perimed, Stockholm, Sweden). The mice's nape fur was shaved and disinfected before being placed under a dissecting stereo microscope (Leica S8 APO; Leica Microsystems, Wetzlar, Germany). An incision of 1–2 cm was made on the clean skin, and margins were pulled laterally to reveal the cranium. A 0.5-mm diameter microfiber laser Doppler probe (Master Probe 418-1 connected to microtip: MT B500-0L240) was attached to the skull with cyanoacrylate glue: 6 mm lateral and 2 mm posterior to bregma. Data on rCBF was expressed as mean percentage of the baseline pre-ischemia value.

The animals were placed supine under the dissection microscope, and the surgical region of the neck was shaved and disinfected. To expose the right common carotid artery (CCA), a midline neck incision was made, followed by dissection of the underlying nerves and fascia. The CCA was carefully separated from the lateral vagus nerve, with special care taken not to damage, stimulate, or puncture it with surgical tools. By blunt dissection of the surrounding tissues, the right external carotid artery (ECA) and internal carotid artery (ICA) were also isolated (rupture of the superior thyroid and occipital arteries was avoided). The ECA was then permanently ligated as distal from the CCA bifurcation as possible, and loosely ligated proximal to the bifurcation. Reduction in rCBF was observed at this point. The ICA was clamped with a microvascular clamp, and the CCA was temporarily closed with a silk suture. A small incision was made in the ECA between the silk sutures, and a silicone-rubber-coated 6.0 nylon monofilament (60[21–23]12PK10 or 60[21–23]23PK10; Doccol Corporation, MA, United States) was inserted into the hole and pushed up to the ICA (CCA now open) until the filament was ≈9 mm from the place of insertion (highlighted on filament with a silver sharpie© pen) and until a sudden drop in rCBF was observed. The filament effectively reached the circle of Willis and blocked the middle cerebral artery (MCA) when this change in rCBF was seen. Filament tip diameter selection, depended on the animal's weight, according to Supplementary Table 1. The loose suture around the ECA was tightened around the inserted filament to prevent movement during the occlusion period. The filament was removed after the designated occlusion time, and the proximal section of the ECA was permanently tied. The temporary tie around the CCA was removed (it was only closed when necessary to prevent blood leakage through the ECA hole), and reperfusion was established, as evidenced by rise in rCBF on the laser Doppler. The surgical procedure was considered adequate if 70% reduction in rCBF occurred immediately after placement of the intraluminal suture and if reperfusion occurred after filament withdrawal; otherwise, mice were excluded. Bupivacaine (2 mg/kg in 0.9% NaCl) was injected into the open wound in the neck of mice. Both the neck and head incision sites were sutured, after the laser Doppler probe was cut close to the skull. As a control, sham-operated animals were subjected to the same procedure without the MCA being occluded. Within the first 24–48 h of surgery, one mouse from the tMCAO 45 min group and one from the tMCAO 25 min group died. Mortality rate in the tMCAO sham group was zero; in the tMCAO 45 min group, it was 16.67%; and in the tMCAO 25 min group, it was 20% at 7 days post-surgery. According to our experience, 2 researchers are required to perform the surgery in 5–7 animals/day (both sham and tMCAO), considering the post-stroke care optimized to reduce mortality and infarct variability. Moreover, considering behavioral evaluation, which includes presurgical training (4 days before) and post-stroke functional evaluation (until 7 days after surgery), we were able to perform surgery on 15 animals per month.

### Postoperative Care (Normal vs. Optimized)

The normal postoperative care used was based on the tMCAO surgery literature's standard postoperative care. It consisted of: controlling mouse's pain with buprenorphine for 8 to 12 h after surgery, placing mashed pellets on the floor of the cage after surgery, and twice-daily subcutaneous fluid supplementation with 5% glucose and 50% Duphalyte (NaCl solution).

For the second animal cohort, an optimized postoperative protocol was designed based on recommendations by Modo et al. (14) and Ryan et al. (17), as well as the IMPROVE guidelines (14, 17, 24) and the expertise of the veterinary responsible for the animal facility.

For the mice to get used to a new object/food, mashed pellets and nutritionally fortified water gel (DietGel Recovery, ClearH2O, Maine, United States) were placed on the cage floor, easily reachable, 1 week prior to surgery.

Each mouse received 0.5 ml saline (0.9% NaCl) and.08 mg/kg buprenorphine subcutaneously for fluid replacement and analgesia immediately after surgery. For the first 3 days after ischemia, buprenorphine was administered at time intervals of 8–12 h, and 0.3 ml of both 5% glucose and 50/50% Duphalyte (NaCl solution) were administered twice a day.

The mice were weighed every day and syringe-fed with 3-4 drops of a fortified food supplement (Anima Strath, Lisboa, Portugal) twice daily. These husbandry practices were continued until animals' weight stopped dropping or until the experiment ended. Daily body weight changes were expressed as percentage of baseline body weight, which was measured immediately before surgery.

After surgery, the mice were immediately placed in a recovery box (small animal recovery chamber/warming cabinet; Harvard Apparatus) at a humidified temperature of 35°C until they fully recovered from anesthesia (~2 h). Once the anesthesia worn off, recovery box temperature was reduced to 33° for the next 12 h; afterwards the home cages were half-placed on a heating pad on the first post-surgery day, allowing the mice to choose their environment during recovery and, thus, control post-stroke hypo/hyperthermia.

To help reduce stress, elements from the home nest such as paper and roll papers were placed in the recovery box. The mice were paired with their pre-surgery cage mates when they were returned to their home cage. However, and because of differences in alertness and motile/cognitive deficits among the animals, the sham-operated animals were separated from the tMCAO-operated animals to avoid stress and fighting in the cage.

Animal humane end point (HEP) was defined for animals that exceeded 25% loss of baseline weight or for animals that exceeded 20% loss of baseline weight and presented elevated scores in terms of appearance and functional parameters, which were evaluated daily using a surgery score sheet.

The sham animals were subjected to the same postoperative protocol as the tMCAO animals.

### Modified DeSimoni Neurological Score

Each mouse was rated on neurologic function scales 2 h after reperfusion using a modified DeSimoni functional score (25, 26). General and focal deficits in the mice are represented by the sum of the score in 13 categories that ranges from 0 (healthy) to 39 (worst performance in all categories). The higher the overall score, the more severe the observed deficit. The mice were assessed for hair (0–2), ears (0–2), eyes (0–3), posture (0–3), and spontaneous activity (0–3), as well as for body symmetry (0–2), gait (0–4), cycling behavior (0–3), forelimb symmetry (0–4), compulsory circling (0–3), climbing on a surface held at 45° (0–3), gripping (0–3), and whisker response to light touch (0–4). This test was performed to confirm model success. Scoring was performed by a trained investigator who was blind to the experimental conditions. Each test was filmed for further analysis.

### Functional Tests

**Figure 1:**
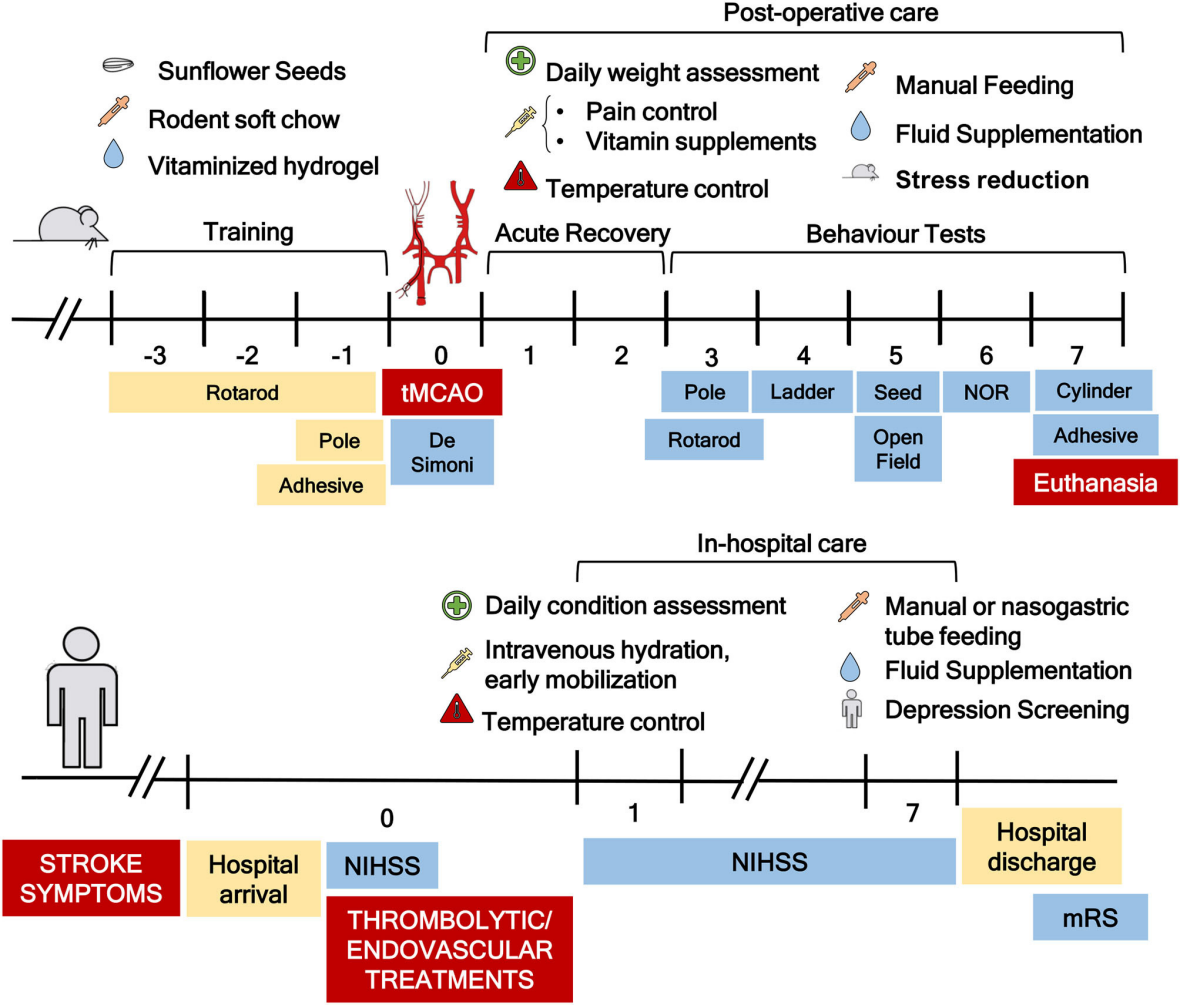
Schematic representation of the timeline, postoperative care protocol, and functional workflow optimized. Prior to transient middle cerebral artery occlusion (tMCAO) surgery, the mice were trained on the rotarod, pole, and adhesive tests. Soft rodent chow, sunflower seeds, and vitaminized hydrogel were also placed on the floor of the cage during that period to allow for habituation. On the day of surgery, DeSimoni neurologic score was used to confirm ischemia induction and evaluate its extension. After surgery, the mice were subjected to a detailed postoperative care protocol that included pain, temperature, and stress control, manual feeding, and fluid supplementation, similar to what is applied to patients with stroke in hospital acute care. Following a period for acute recovery of 2 days, the mice were subjected to the schematized functional workflow. In the bottom part of the figure, Stroke patients therapeutic workflow is represented. A clear similarity between mice and patients can be observed.

Mice from the second cohort were subjected to a battery of nine sensorimotor tests from day 3 to day 7 post-tMCAO according to the experimental design shown in Figure 1. An investigator who was blind to the experimental conditions conducted the tests in the dark (active) phase. Between animals, materials were cleaned with an odorless neutral detergent. All of the mice were divided into groups in random and were given numbers to identify them. To allow for precise posteriori software/manual quantification, all behavior tests were videotaped. The event-logging behavior quantification software BORIS (Behavioral Observation Research Interactive Software, Torino, Italy) (27) or the tracking system SMART (Smart Video Tracking Software v3.0; Panlab, Spain) were used to quantify the evaluation parameters of each test. When software-based semi-automatic quantification was not possible, manual quantification was performed.

### Pole Test

The pole test was performed to assess the mouse's motor coordination, as previously described (28–32), with minor modifications. A vertical wooden pole (50 cm in length, 1 cm in diameter) with a rough surface was placed inside a type III cage as the test apparatus. A mouse was placed in the pole with its head up, and its descent was recorded. The time the mouse took to turn head downward [time to turn (in s)] and the total time it took to descend to the cage floor [time to descend (in s)] were measured. The maximum test time was set at 120 s. If the mouse descended partway but still fell, the behavior was scored until the moment it reached the floor. However, when the mouse was unable to turn but, instead, fell from the pole, the time to descend was measured as 120 s. Mice that did not turn but descended in a lateral position were excluded. The animals were trained to perform the task for one session before surgery (D-1) and tested three times after surgery (D+3). Inter-test interval was 5 min. Scores from the three trials were averaged for analysis.

In this experiment, one mouse was unable to turn and slid down the pole laterally and was excluded.

### Accelerated Rotarod

Motor coordination and balance were determined with an accelerated rotarod, which was used as previously described (32, 33) but with minor alterations. The rotarod apparatus was made up of a black striated rod (with a diameter of 3 cm) divided in 5 compartments (with 5-cm width), at a height of 20 cm from tilting planks (TSE Systems, Bad Homburg, Hessen, Germany). Before surgery, mice were trained to balance on the rotating rod for 2 days (D-3 and D-2). On the first day of training, they were placed on the apparatus for 1 min without rotation and then for 2 min with constant low-speed rotation (4 rpm). If a mouse fell from the rotating rod, it was repositioned until it could stay for at least 2 min. On the second day of training, the mice were subjected to the accelerated protocol (4–40 rpm, 2 min) until they stayed in the apparatus for at least 60 s. On the day before surgery (D-1), a baseline assessment was performed. The mice were tested on day 3 (D+3) post-surgery: each mouse was placed on the rod rotating from 4 to 40 rpm over 2 min. Maximum test duration was set at 120 s. The time each mouse was able to walk on the rod before falling was recorded in s (latency to fall). Occurrence of two consecutive passive rotations, i.e., if an animal gripped the rung and spun two consecutive revolutions rather than actively walk, was considered a fall. All the mice were tested three times, with an inter-test interval of 5 min. Mean latency to fall from the three trials was used for data analysis.

### Ladder Rung Walking Test

In the ladder rung walking test, the animals were scored for their ability to cross a 60-cm long horizontal metal-rung ladder with 1.5 cm gaps to assess motor coordination and skilled walking (34, 35). The ladder was elevated 25 cm above the ground, with a refuge (home cage) at the end of the ladder and a new cage at the beginning. To prevent the animal from turning around, the width of the alley was adjusted to the animals' size. The mice were placed on one side of the ladder and made to cross to the other. The animals were tested in five trials on day 3 post-surgery (D+3), with a 5-min -trial interval. The placement of each forelimb and hindlimb on the rungs was qualitatively quantified using the 7-category scale described in (36), with foot placement on the rung being rated according to its position and placement accuracy/errors. Only consecutive steps were analyzed; as such, the last step before a stop and the first step after the stop were not scored, and the last stepping cycle at the end of the ladder was also excluded. The lowest score was recorded when multiple errors occurred at the same time. The number of errors for each crossing was also recorded. An error was defined as limb placement that received a score of 0, 1, or 2 points on the foot fault scoring system. For each limb, the number of errors and steps was recorded separately. When the forepaw was correctly placed on the rung, i.e., when the placement score was 6, digit score was also measured in a three-s scale in order to evaluate the degree of digit flexion around the rung. Scores from the five trials were averaged for analysis.

### Open Field Test

The open field test, with few modifications from that described in (37, 38), was performed to examine spontaneous locomotor activity, as previously described in (37, 38) but with minor revisions. Each mouse was placed in the center of an opaque arena (43 cm × 43 cm with 45-cm high walls) on day five post-surgery (D+5) and allowed to move freely for 15 min. The number of rearing and grooming episodes was recorded, as well as the direction of each turn a mouse took. Total distance traveled, mean walking speed, peripheral activity (locomotion along the walls), and center activity (locomotor activity in the central zone) were automatically obtained through video tracking.

### Novel Object Recognition

The novel object recognition (NOR) test was performed to examine alterations in recognition memory, which is a hippocampal-dependent working memory (38–40). The NOR test was conducted in an arena (43 cm × 43 cm, 45-cm height) and consisted of three phases: the 15-min habituation phase, in which the mice were allowed to explore the apparatus freely; the 10-min acquisition/sample phase, 24 h after habituation, where the mice were placed in the apparatus with two identical plastic objects (familiar object); and the 3-min retention/choice session performed 4 h after the acquisition phase and in which one of the familiar objects was substituted by a novel object, and the mice were able to explore both objects. The objects chosen for this experiment were of similar height and weight but varied in shape, color, and materials (Legos, glass cups, or toys). The habituation phase was performed 5 days after surgery (D+5) during the open field test, since the arena is the same, and the acquisition and retention phases were performed on day 6 post-surgery (D+6).

Object exploration was defined as a mouse's snout touching the object or directed towards the object at a distance of less than 2 cm. Exploratory behavior did not include climbing on or rearing near an object. Time spent exploring each object was recorded, and discrimination index (DI) was calculated as the ratio (T_N_-T_F_)/(T_N_+T_F_), with T_N_ representing the time an animal spent exploring the novel (N) object and T_F_ the time the same animal spent exploring the familiar (F) object in the choice phase. Mice that spent periods lower than 1 s exploring both objects (total exploration time) in the choice session were excluded, resulting in exclusion of 8 mice (4 from WT and 4 from tMCAO).

### Sunflower Seed Challenge

The sunflower seed challenge was adapted from rat to mice (6). The mice were given sunflower seeds in their home cages in the week leading to surgery to get them used to shelling and consumption of seeds. On day 5 after surgery (D+5), the mice were placed in a type II cage deprived of all bedding and given two sunflower seeds. After 30 min, the number of whole (uneaten) seeds and pieces of shell were counted. The animals were not fasted prior to testing. The mice were tested on a single trial.

### Cylinder Test

Paw preference in weight-bearing was evaluated using a transparent cylinder (41–43)_7 days after surgery (D+7). The mice were placed in a transparent cylinder (10-cm diameter and 15-cm height) and video-recorded for 10 min. The forelimb used to make contact with the wall, during rearing or during lateral exploration was appointed by the following criteria: (1) the first forelimb to contact the wall during a full rear was recorded as an “independent” wall placement for that limb; (2) simultaneous use of both forelimbs in wall contacting during a full rear or lateral movement along the wall was recorded as movement of “both” forelimbs; (3) if after the first forelimb made contact to the wall, the other forelimb was also placed on the wall, but the first was not removed at any moment, an “independent” movement for the first forelimb and a “both” movement were recorded; (4) when the mouse explored the wall laterally alternating between both forelimbs, it was recorded as one “both” movement. For an animal to receive another wall movement score, both paws had to be removed from the vertical surface. A total of 20 movements were recorded during the test. The videos obtained were used a *posteriori* by a blinded experimenter for analysis.

Behavior was expressed as a single overall limb use asymmetry score and calculated as described in (44): asymmetry score = $\frac{I\;-\;C}{I+C+B}$, with “I” being the number of ipsilateral movements, “C” the number of contralateral movements, and “B” the total number of movements.

### Adhesive Removal Test

The adhesive removal test (also known as the sticky tape test) is performed to assess forepaw sensitivity and somatosensory neglect, as well as motor impairments after stroke (6, 32). Before each testing session, the mice were placed in a transparent type II cage with no bedding for a 60-s habituation period. Following that, two adhesive tapes of equal size (0.3 cm × 0.4 cm) but with two different colors were applied with equal pressure on each paw of the animals to cover the hairless part of the forepaws. The adhesives had different colors to make video quantification more straightforward. Each trial and animal had a different order in which the adhesive was applied, and each forepaw was touched firmly and quickly after the tape was applied. The mice were then placed in the type II cage. The time it took to make contact and remove each adhesive tape was recorded, with a maximum test time of 120 s. Three trials were undertaken with 1-min inter-trial interval, and time from the trials were averaged for analysis. Baseline behavior of the mice was recorded the day before surgery (D-1), and they were tested 7 days post-surgery (D+7). The trials were videotaped from below the transparent cage so that the mice's limbs could be observed during the recording.

### 2,3,5-Triphenyltetrazolium Chloride Staining

On day 7 post-tMCAO, animals from the first cohort were euthanized with a mixture of ketamine (170 mg/kg) and medetomidine (2 mg/kg) (5:1, in 6,5 ml of, 9% saline solution), and infarct volume was evaluated by 2,3,5-triphenyltetrazolium chloride (TTC) staining, as previously described in (45) but with minor changes. Briefly, the brain was removed, and the forebrain was sliced into 1-mm-thick sections using a mouse brain slicer (Acrylic Brain Matrix, Coronal, 40–75 g, Stoelting, Wood Dale, IL, United States) on ice. The sections were first rinsed in ice-cold 0.9% sodium chloride (NaCl) for 10 min before being immersed in 25 ml of 1% TTC (Sigma-Aldrich, St. Louis, MI, United States) in 0.9% NaCl at 37°C for 15 min. Then, the slices were rinsed again in 0.9% NaCl, and images were taken with a camera and analyzed *a posteriori*. The section's unstained area was regarded as an infarct area, whereas the stained area was classified as a non-infarct area. Infarct volume was determined with a semi-automated quantification method in Fiji-ImageJ (46). To begin, the images were calibrated with a millimetric piece of paper that was included in all photos next to the slices as a reference. Next, the images were batch-processed and analyzed in ImageJ using a custom-made macro. Each image, containing multiple slices of the same condition, was pre-processed to isolate each slice. Next, a manual selection of a color threshold was done, followed by a Gaussian filter (sigma 10). Slice segmentation was performed with the threshold Huang's method, and adjusted using morphometric operations fill holes and dilation. The output obtained in this pre-processing step is the centroid of each slice. Finally, each slice was segmented alone by duplicating a rectangle centered in the detected centroids, with size *w x h* (*w* = original image width, and *h* = original image height/number of slices).

Infarct lesion measurement of each slice was performed by a blind experimenter with the following steps: (i) manual delineation of the middle line to divide the slice into two hemispheres; (ii) manual annotation of stroke area (if present); and (iii) quality control of resulting regions of interest. The macro produces a results table with slice labels, the contra- and ipsilateral hemisphere areas, and the area of infarct lesion. Volumes were calculated by summing the areas from all the brain slices (considering that each slice's thickness is 1 mm).

Infarct volume was calculated using correction for edema and/or brain atrophy by applying the equation:


$$\begin{array}{l} \text{Total lesion }\left(\text{\% of total brain}\right)\;=\frac{\text{Lesion}*\frac{\text{Contralateral hemisphere}}{\text{Ipsilateral hemisphere}}}{\text{Total brain}}\\ \;*100 \end{array}$$

with lesion, contralateral hemisphere, and ipsilateral hemisphere being the volumes obtained for each of these parameters, respectively, in μm^3^, during image quantification and based on (47).

### Statistical Analysis

Preliminary power calculations were not performed. Statistical analysis was performed using GraphPad Prism Version 8.0/9.0 (GraphPad Software, Inc., San Diego, CA, United States). Supplementary Table 3 provides a detailed description of statistical results of all the data. The data were collected from experiments and presented as mean ± SEM, with individual points. The experimental unit is each animal, and the number of animals used in each experiment/group is described in figure legends. Normality tests were performed (from Anderson-Darling to Shapiro-Wilk tests) and indicated normal/Gaussian distribution; thus, a parametric statistical analysis was adopted. The statistical analysis was performed by one-way analysis of variance (ANOVA) or two-way analysis of variance (ANOVA), followed by Tukey's multiple comparison test. For all the statistical analyses: ^****^p < 0.0001, ^***^p < 0.001, ^**^p < 0.01, and ^*^p < 0.05; ns means not significant. No analysis was performed to identify and remove outliers.

## Results

### Improving tMCAO Mouse Model to Reduce Variability and Increase Translation

**Figure 2:**
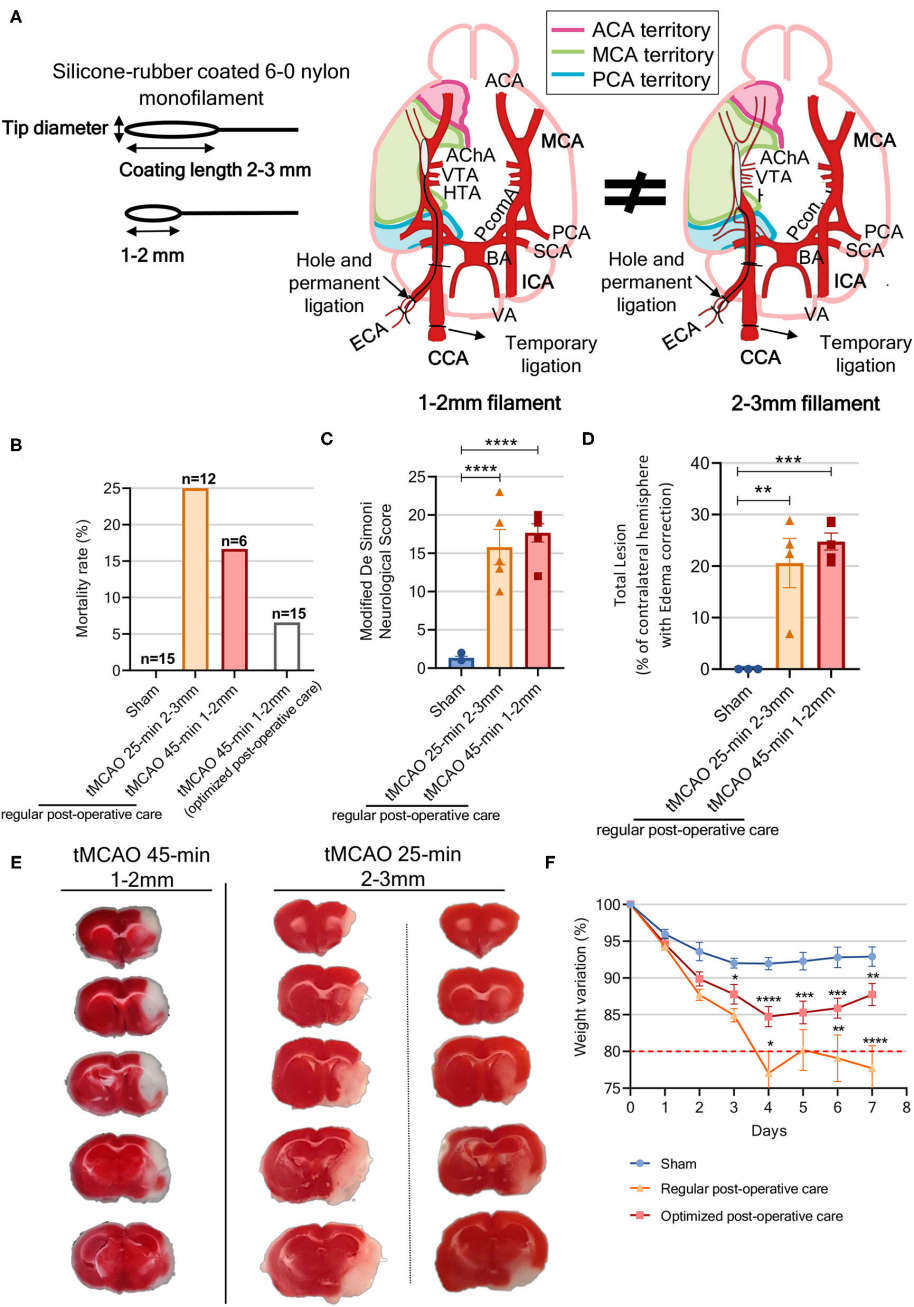
Improving tMCAO surgery parameters and postoperative protocol to shorten the gap between preclinical and clinical stroke studies. **(A)** Schematic of nylon sutures with different silicone coated tip diameters and lengths; suture coating lengths used varied between 1–2 and 2–3 mm, while suture tip diameter varied according to animal pre-surgery weight, and schematic diagram of different arteries that become occluded with different suture types, which lead to different territories being differently affected depending on the main arteries' irrigation territory. **(B)** Mortality rate of the different groups (*n* = 6–15). **(C)** Neurologic score severity as evaluated by the modified DeSimoni scale [sham *n* = 6 (1 male, 5 females), tMCAO 25 min *n* = 5 (4 males, 1 female), tMCAO 45 min *n* = 6 (3 males, 3 females)]. **(D)** Lesion volume calculated from brain slices was corrected for edema and/or brain atrophy and presented as percentage of total brain volume [sham *n* = 3 (1 male, 2 females), tMCAO 25 min *n* =4 (3 males, 1 female), tMCAO 45 min *n* = 5 (3 males, 2 females)]. **(E)** Representative photography of TTC-stained brain slices from the tMCAO animals whose occlusion parameters were 45-min occlusion with 1- to 2-mm filament (left panel) and 25-min occlusion with 2- to 3-mm filament (right panels). **(F)** Bodyweight loss post-MCAO was assessed daily for a 7-day period in animals that were subjected to both the normal and the optimized postoperative care: sham with optimized postoperative care, *n* = 10 (3 males, 7 females); tMCAO with normal postoperative care (NPC) (tMCAO 25-min *n* = 9, tMCAO 45-min *n* = 6) *n* = 15 (8 males, 7 females); tMCAO with optimized postoperative care (OPC) (tMCAO 45-min) *n* = 13 (7 males, 6 females). Statistics refer to sham vs. OPC and OPC vs. NPC ****p < 0.0001, ***p < 0.001, **p < 0.01, *p < 0.05. **(C,D)** One-way ANOVA and **(F)** two-way ANOVA followed by Tukey's multiple comparison *t*-test. ACA, anterior carotid artery; AChA, anterior choroidal artery; BA, basilar artery; CCA, central carotid artery; ECA, external carotid artery; ICA, internal carotid artery; HTA, hypothalamic artery; MCA, middle cerebral artery; PCA, posterior cerebral artery; PcomA, posterior communicating artery; SCA, superior cerebellar artery; VA, vertebral artery; VTA, ventral thalamic artery.

In order to achieve a reproducible stroke infarct region using the intraluminal mouse stroke model, we investigated the effects of applying two different commercial suture coating lengths (1–2 vs. 2–3 mm) (Figure 2A) combined with two different occlusion times (25 vs. 45 min). Longer coating filaments and longer occlusion times are usually associated with larger infarct volumes (48, 49); however, homogenous parameters are absent in the literature. Regarding mortality rate, we observed no mortality in the sham group, while the tMCAO groups presented 16.7 and 25% mortality rate 7 days after the surgery for the 45 min group (1–2 mm coating length) and the 25 min group (2–3 mm coating length), respectively (Figure 2B).

Size-matching of animal and suture (Supplementary Table 1) was reported to be an important feature to produce consistent cerebral infarction (50, 51), since too small sutures lead to insufficient ischemia (incomplete vessel obstruction), while too large sutures either do not pass through the vessels or cause intracerebral hemorrhage according to our experience in this and other animal cohorts.

Two hours post-reperfusion, neurological evaluation was performed to confirm occlusion success using the modified DeSimoni neurological score. The sham group scored 1.33 ± 0.2 (corresponding to no deficit), whereas the tMCAO-45 min and 25 min groups scored 17.67 ± 1.2 and 15.8 ± 2.3, respectively, demonstrating occlusion success (sham vs. tMCAO 25 min, *p* < 0.0001; sham vs. tMCAO 45 min, *p* < 0.0001) (Figure 2C).

Brain infarct was evaluated by TTC staining, with both tMCAO occlusion times inducing extensive damage to the brain's right hemisphere, whereas the sham animals presented no lesions. In the tMCAO45 min group, a lesion occupied 24.8 ± 1.7% of the whole brain and included the majority of ipsilateral somatosensory and motor cortices, as well as a large portion of the striatum (Figures 2D,E). However, in the tMCAO 25 min group, and despite a mean lesion of 20.6 ± 4.7% of the whole brain, lesion morphology was more variable, and could include either a part of the motor and somatosensory cortices and the striatum, only the cortex, or only the striatum (Figure 2E).

In preliminary results, when surgery was performed by combining a 1-to 2-mm filament with a 25-min occlusion, no lesion was observed (Supplementary Figure 1A), while when surgery was performed with 2- to 3-mm filaments and 45-min occlusion, other brain regions outside the MCA territory, such as the hippocampus and hypothalamus, were also affected (Supplementary Figure 1B). Taken together, these results indicate that the combination of 1-to 2-mm filaments with a 45-min occlusion leads to consistent and less variable brain lesions in mice subjected to tMCAO.

### Establishment of Extensive Postoperative Care Similar to In-hospital Care of Patients, Increases Animal Wellbeing and Decreases Mortality

To improve animal welfare, decrease mortality, and establish a clear parallel between patients with clinical stroke and the preclinical stroke model, we adopted an original postoperative care protocol on mice similar to the one applied to patients. We introduced several important procedures: special feeding with moistened pellets; water/vitamin supplementations [providing vitaminized water gel (oral) and Duphalyte (subcutaneous injection)]; hypoglycaemia control with glucose saline buffers (subcutaneous injection) together with mouth-feeding of a highly caloric additive, AnimaStrath; stress reduction by maintaining cage mates (and bed materials from original cage after surgery); and pain control to allow for some self-feeding (Supplementary Table 2).

All animals treated with the optimized postoperative care tolerated the surgery with a lower mortality rate of 6.6% 24 h post-stroke (only 1 tMCAO mouse died, *n* = 15), instead of 17% when the regular postoperative care protocol was used.

To monitor the success of the implemented postoperative care, weight loss was compared between mice subjected to the 45-min occlusion with 1- to 2-mm filament, treated either with our improved postoperative care protocol or with the regular postoperative protocol (Supplementary Table 2). The regular postoperative care group showed a weight reduction of more than 20% from baseline, whereas the optimized postoperative care group presented only 10% weight loss, which was closer to that of the sham mice (Figure 2F). Moreover, some animals in the regular postoperative care group came dangerously close to the humane end point parameter, set as 20–25% weight loss. Thus, application of the improved postoperative care protocol described here improves animal welfare, reduces mortality rate (by ~60%, from 17 to 6.6%), and allows for better assessment of therapeutic strategies, since the animals are not in a life-threatening end point.

### tMCAO Model Induces Motor Deficits in Mice Similar to Patients With Stroke

In order to establish a complete functional evaluation to assess stroke effects on mice, eight sensorimotor and cognitive tests (Figure 1) were selected, implemented, and scheduled according to the literature, pilot studies, and clinical practice (NIHSS and mRS). The designed workflow of functional tests considered several important factors to ensure its applicability, particularly appropriate number of test(s)/duration per day, animal recovery/motivation, potential interplay between tests, and animal stress. It was applied to the control, sham-operated and stroke animals (45 min, 1-to 2-mm filament, optimized post-stroke care) to assess its reliability and sensitivity in assessing stroke effect.

**Figure 3:**
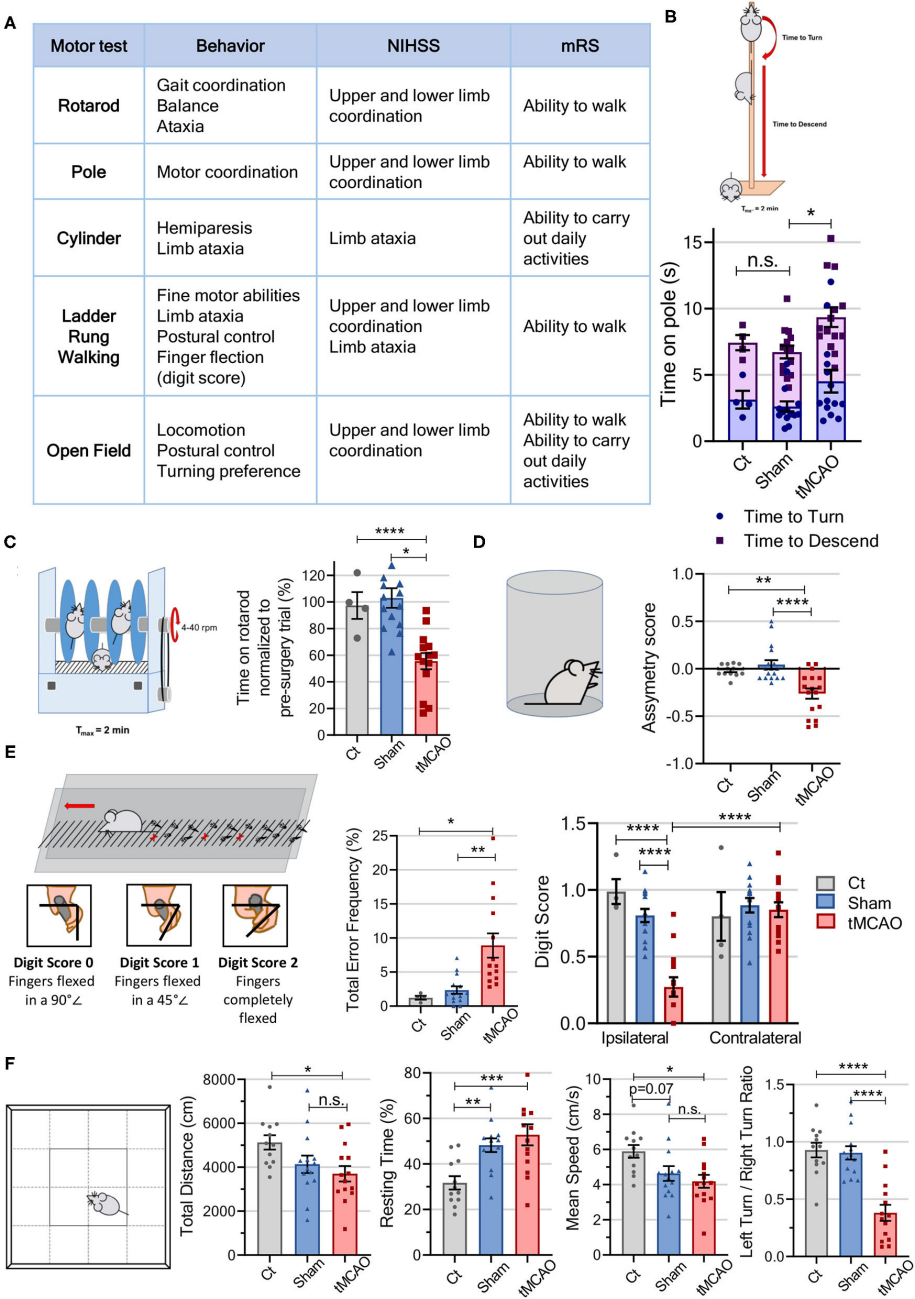
Motor tests of the behavior workflow allow for differentiation of tMCAO from sham and Control animals in different locomotor tasks. **(A)** Table comparing the parameters evaluated in the motor tests to the scores assessed in both the mRS and the NIHSS. **(B)** Schematic representation of the pole test. Time, in seconds, the mice took to both turn and descend a vertical pole presented in the graph [CT *n* = 4 (4 females), sham *n* = 14 (5 males and 9 females), tMCAO *n* = 14 (9 males and 5 females); statistical meaning over time to descend]. **(C)** Schematic representation of the rotarod test. The time the mice spent on the rotarod post-surgery was normalized to baseline values and presented in the graph [CT *n* = 4 (4 females), sham *n* = 14 (5 males and 9 females), tMCAO *n* = 14 (9 males and 5 females)]. **(D)** Schematic representation of the cylinder test. Cylinder's asymmetry score (difference between ipsilateral and contralateral overall limb use) allows to determine of which paw the mice preferentially use while weight-bearing [CT *n* = 12 (8 males and 4 females), sham *n* = 17 (5 males and 12 females), tMCAO *n* = 17 (9 males and 8 females)]. **(E)** Schematic representation of the ladder rung crossing and digit score evaluation. The ladder rung allows for evaluation of mice's misplacement of the paws (error frequency as a percent of total steps) and fine motor abilities (digit scores from 0 to 2) [CT *n* = 4 (4 females), sham *n* = 14 (5 males and 9 females), tMCAO *n* = 14 (9 males and 5 females)]. **(F)** Schematic representation of the open field arena. The open field paradigm allows for discrimination of mice locomotor gait [distance, resting time, speed, and turn propensity (left/right turn ratio)] [CT *n* = 12 (8 males and 4 females), sham *n* = 14 (5 males and 9 females), tMCAO *n* = 14 (9 males and 5 females)]. ****p < 0.0001, ***p < 0.001, **p < 0.01, *p < 0.05. One-way ANOVA or two-way ANOVA followed by Tukey's multiple comparison *t*-test.

In clinics, patients show decreased postural control and balance deficits, which increase their risk of falling. These deficits were tested on the mice using the pole (28, 30, 31) and the accelerated rotarod tests (10, 52). In the pole test, the tMCAO group significantly differed from both the control and sham groups in time to descend, taking almost twice as long, as well as in time to turn, with the tMCAO group taking 50% more time to turn (*p* = 0.08). Importantly, the sham animals did not differ from the control mice, indicating that the effect was stroke-specific (Figures 3A,B; Supplementary Video 1). The performance of tMCAO mice on the accelerated rotarod displays a significant decrease (~40%) in time spent on the rod when compared to the control or sham animals (Figure 3C).

Other relevant stroke deficits observed in patients are hemiparesis on the ipsilateral side of the body and limb hemiplegia. Mouse-related deficits can be observed performing both the cylinder and ladder rung walking tests (13, 36). The cylinder test allows for evaluation of limb hemiparesis, assessed with the cylinder asymmetry score. This test shows that the tMCAO mice use their contralateral paw 20% more than the control groups (Figure 3D; Supplementary Video 2). In the ladder rung walking test, which allows for evaluation of fine motor skills, the tMCAO animals showed a significantly higher error frequency (3 times higher) than the control and sham groups (Figure 3E; Supplementary Video 3). Importantly, this skilled walking task allows for evaluation of each limb separately (34, 53), and we observed that tMCAO animal's ipsilateral forepaw positioning is impaired, as evaluated with the digit score, when compared to the control and sham mice, as well as to their own contralateral paw. In contrast, for the contralateral paw, no differences were observed among the groups. The ladder rung test also allows for a seven-category classification of each step based on limb placement on the rung (Supplementary Figures 2A,B), with more than 50% of forelimb steps in the control and sham groups classified as “correct placement,” whereas for the tMCAO animals, more than 50% of steps were classified as “partial placement” and “slight slip.” Additionally, we observe that only the ipsilateral forepaw is affected, when comparing the control and sham groups with the tMCAO mice (Supplementary Figure 2B).

Decreased postural control, balance deficits, and hemiparesis lead patients to demonstrate asymmetrical gait patterns and decreased walking speed (54). Mouse-related deficits in locomotor activity can be measured in the open field arena, with stroke mice displaying a significantly decreased locomotor activity, walking 30% less (control vs. tMCAO) at a 30% lower speed, and spending 40% more time resting than the control animals (Figure 2F). However, no significant differences between sham and tMCAO were observed, indicating that the surgery is a preponderant factor to take into account regarding locomotor deficits. The tMCAO animals also presented a significantly decreased tendency to turn to their left/affected side, turning to the right twice as much as they turned left, while the control groups (control and sham) made equal turns to both sides, meaning laterality is a relevant measure to observe stroke effects by open field test.

In the clinic, patient strength and grip are also evaluated. These parameters were evaluated on the mice by wire hanging gripping test, but no differences were found between the tMCAO and sham groups (Supplementary Figure 3). The test was removed from the workflow.

### tMCAO Model Induces Sensorimotor Deficits in Mice Similar to Patients With Stroke

**Figure 4:**
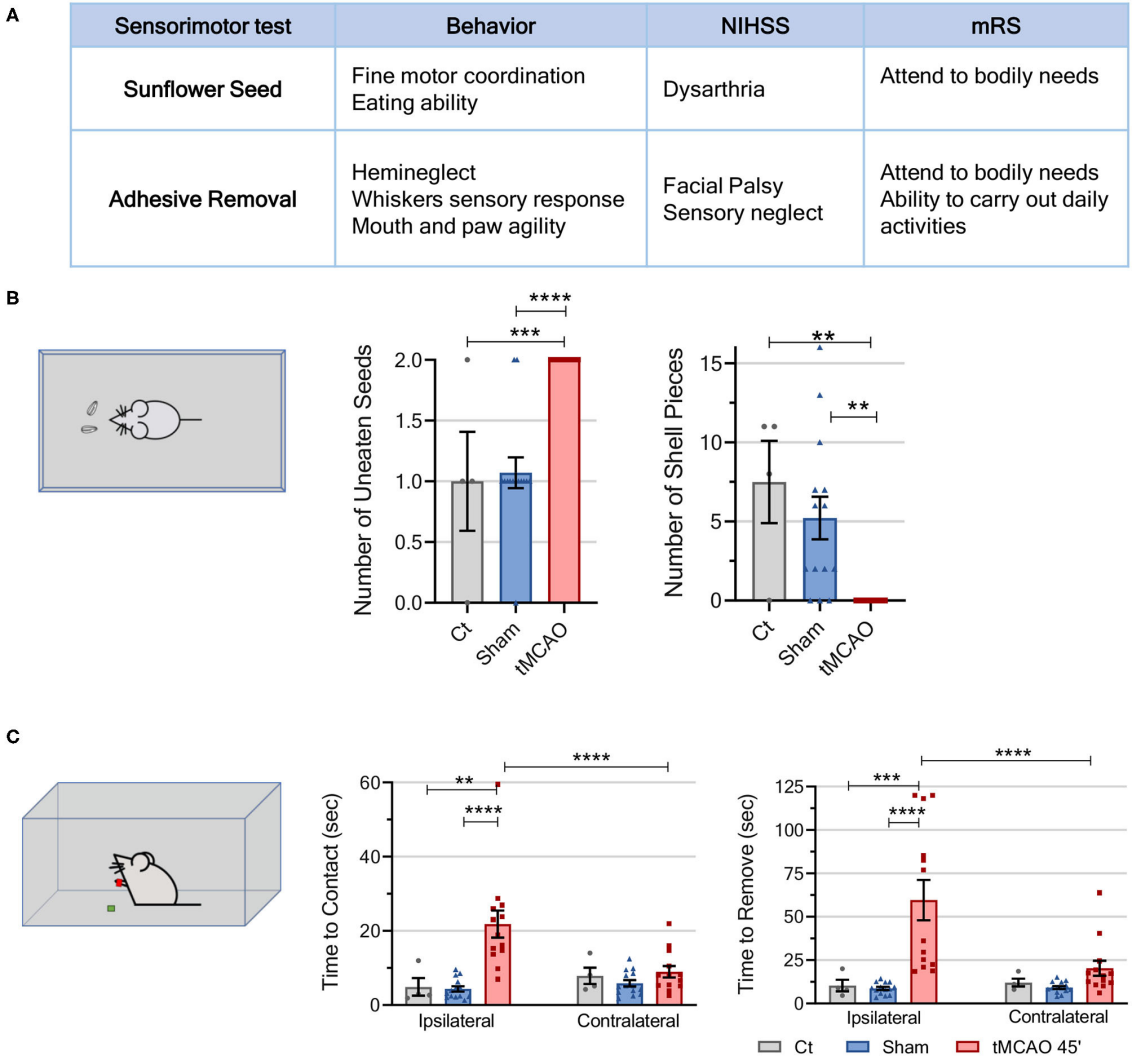
Sensorimotor tests from the behavioral workflow display tMCAO's sensorial and fine motor coordination deficits. **(A)** Table comparing the parameters evaluated in the sensorimotor tests to the scores assessed in human patients using the mRS and NIHSS. **(B)** Schematic representation of the sunflower seed challenge. The sunflower seed challenge allows for differentiation of sensory and fine motor impairments in stroke mice by evaluating the number of unopened seeds and shell pieces produced by the animals in a 30-min period of time [CT *n* = 4 (4 females), sham *n* = 14 (5 males and 9 females), tMCAO *n* = 14 (9 males and 5 females)]. **(C)** Schematic representation of the adhesive removal test. Sensorimotor deficits can be observed in the adhesive test through the time, in seconds, the mice take to make contact and remove the adhesive from either paw. A delay in sensing and making contact with the adhesive in the paw demonstrates sensorial deficiency, and the lingering time to remove it demonstrates facial palsy and more motor deficits [CT *n* = 4 (4 females), sham *n* = 14 (4 males and 10 females), tMCAO *n* = 13 (8 males and 5 females)]. ****p < 0.0001, ***p < 0.001, **p < 0.01, *p < 0.05. One-way ANOVA or two-way ANOVA followed by Tukey's multiple comparison test.

Hemiparesis and neuromuscular incoordination compromise the engagement in normal activities of patients with stroke, especially regarding reaching and handling objects, which require fine motor coordination. This complex sequence of movements can be observed in rodents when eating special foods such as sunflower seeds (13, 32). Besides, since dysphagia affects 30–64% of patients in the acute phase of recovery, this test also allows for observation of mice's eating ability. The tMCAO mice subjected to this test display an evident difficulty in manipulating and eating the seeds, since no animal was able to eat any seeds (no pieces of shells were present), in clear contrast with the control/sham mice, which ate at least one seed (and produced a high number of shells pieces) (Figures 4A,B).

Patients with stroke usually present simultaneous extinction as a chronic residual deficit, which impairs their ability to perceive multiple stimuli of the same type (13, 32, 55). This phenomenon can be studied on rodent models by adhesive removal test (Figure 4A; Supplementary Video 4). The “time to contact” parameter evaluates forepaw sensitivity and demonstrates sensory deficits. Concordantly, it took the tMCAO animals, on average, 15 s longer (a 400% increase) to make contact with the adhesive on their ipsilateral paw than the sham or control animals, which presented similar times (Figure 4C). Concerning contact of the contralateral paw with the adhesive, the groups did not differ. Additionally, time to remove the adhesive demonstrates both sensorial and fine motor coordination deficits, with the tMCAO animals taking 80 s longer (a 500% increase) to remove the adhesive from the ipsilateral paw. No differences were observed in time to remove the adhesive from the contralateral paw (Figure 4C).

In summary, the workflow of functional tests developed in this study was effective in evaluating sensory and motor disabilities, as well as fine motor skills in the context of daily life activities, triggered by the tMCAO mouse stroke model, similar to what is observed in patients.

### tMCAO Model Induces Cognitive Deficits in Mice, Similar to Patients With Stroke

**Figure 5:**
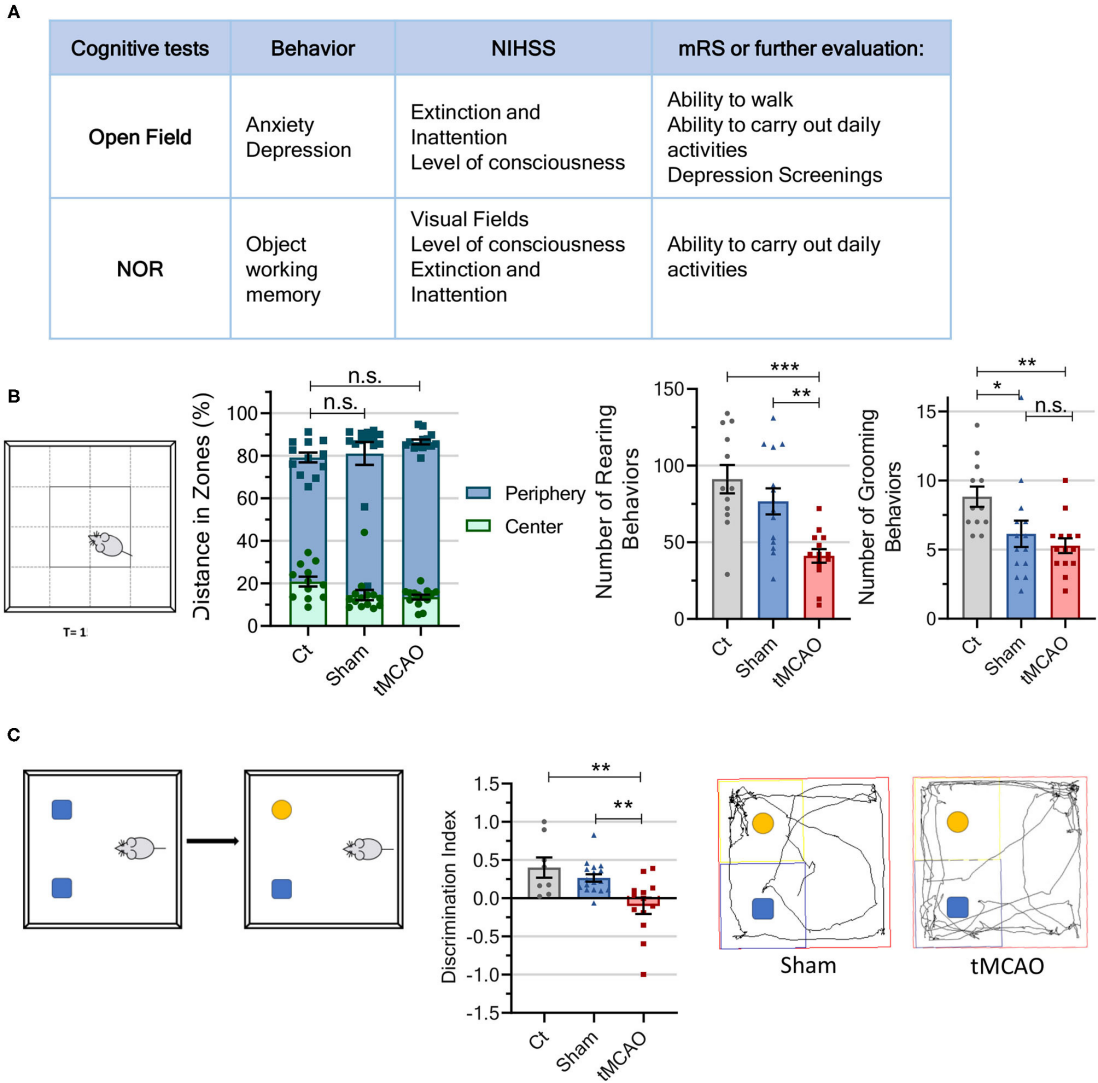
Cognitive tests from the behavioral workflow indicate that subjecting mice to a surgery procedure may increase anxiety-like behaviors and that ischemic lesion leads to memory loss. **(A)** Table comparing the parameters evaluated in the cognitive tests to the scores assessed in human patients using the mRS and NIHSS. **(B)** Schematic representation of the open field arena. Besides the previously mentioned motor parameters, the open field also allows for determination of mice's exploratory behavior. When the mice move more along the arena walls (percent of distance traveled in each arena zone, center and periphery) and perform fewer grooming behaviors, it points to increased anxiety, while lack of rearing behaviors points to lack of exploration [CT *n* = 12 (8 males and 4 females), sham *n* = 14 (5 males and 9 females), tMCAO *n* = 14 (9 males and 5 females); statistical meaning for both periphery and center]. **(C)** Schematic representation of the novel object recognition test and representative tracing of gait pattern from the tMCAO and sham animals. The NOR test allows for evaluation of memory, since the animals demonstrate memory deficits when they spend the same amount of time exploring the novel and familiar objects, as well as a negative mean discrimination index (time spent exploring novel/familial objects ratio) [CT *n* = 8 (4 males and 4 females), sham *n* = 17 (4 males and 13 females), tMCAO *n* = 13 (5 males and 8 females)]. ****p < 0.0001, ***p < 0.001, **p < 0.01, *p < 0.05. One-way ANOVA followed by Tukey's multiple comparison test.

An increasing interest has been given to post-stroke depression after it was demonstrated that 20-50% of patients with prefrontal cortical stroke experience this symptom (56). The open field test also allows for evaluation of mice's anxiety-like behavior as an indicator of depression (Figure 3F). Decreased time spent in the center of the arena (57), as well as reduced grooming behaviors (58), suggests stress and anxiety-like behavior, whereas number of rearing behaviors is considered a good measure of exploration (59). The tMCAO mice spent 10 s less in the center of the arena and performed half of the rearing and grooming behaviors when compared to the control animals (Figure 5B). In the same way, the sham animals spent 10 s less in the central part of the arena and performed half of the grooming behaviors compared to the control mice. However, the number of rearing occurrences was similar to that of the control mice (Figures 5A,B). Thus, the tMCAO mice seem to be in a different motivational state than the sham mice regardless of motor impairments. The described test shows that the tMCAO and sham animals display anxiety-like behaviors probably related to the surgical procedure.

Cognitive impairments also occur following cerebral ischemia, and are particularly related to spatial and non-spatial memories in both humans and rodents (32, 41). The novel object recognition (NOR) test is performed to examine alterations in recognition memory of rodents, a striatal-/hippocampal-dependent working memory (38–40). This test shows that the tMCAO mice spent nearly the same time exploring both objects (familiar: 3.5 s vs. novel: 3.2 s), while the sham and control animals spent significantly more time exploring the novel object (control: familiar: 2.4 s vs. novel: 9.1 s; sham: familiar: 4.3 s vs. novel: 7.7 s). The tMCAO animals differ significantly from the control mice in discrimination index (*p* = 0.0028), as well as from the sham animals (*p* = 0.0081), while the sham animals did not differ from control. This indicates that the tMCAO mice display memory deficits probably due to alterations in thalamic-cortical circuits after ischemic injury.

## Discussion

In this study, we took into close consideration the clinical reality of patients with stroke to optimize the preclinical model of tMCAO in mice, and to implement an innovative sensorimotor/cognitive functional workflow to better assess stroke outcomes, which will be of major importance to evaluate the impact of future therapeutic strategies. During the surgical procedure, the use of shorter coated filaments combined with 45-min occlusion is clearly the best parameter to obtain a reproducible infarct region without threatening mice's survival. Additionally, optimized post-stroke care, similar to what is established for patients in hospitals, reduces mortality and improves animal welfare, both of which are essential to perform a meaningful battery of functional tests to assess stroke outcomes.

Around half patients with ischemic stroke suffer from MCA stroke that results in a small lesion (4.4–14% of the affected hemisphere) (1, 60), with less than 1% of ischemic strokes affecting a large area of the ipsilateral hemisphere (1). Considering that the primary outcome in most stroke studies involves assessing infarcted tissue volume, the lesion induced by preclinical models should be consistent and representative of the infarct observed in humans. Nevertheless, the intraluminal tMCAO model, the most used in the field, presents a great discrepancy in lesion volume, with occlusion time before reperfusion being the most important parameter to consider (61). Morris et al. (48) described that lesions were noticeable following a 15-min occlusion, and that ischemic volume increased and stabilized after 45 min. Our results follow these conclusions, since the tMCAO 45 min animals showed lesions with reduced variability, affecting both the cortex and striatum, compared to mice submitted to 25-min occlusion, in which lesions ranged from slightly affecting the striatum or cortex to affecting the majority of the hemisphere. Another parameter highly associated with lesion variability is length of monofilament coating. Akamatsu et al. (62) showed that 1- to 2-mm coated filaments resulted in lesions affecting only the MCA territory, whereas 3- to 4-mm coated filaments lead to lesions affecting both the MCA and PCA territories. In that study, they concluded that occlusion within the PCA territory results in higher lesion variability. On the other hand, the high mortality rate associated with the tMCAO model has been previously associated with blockage of the posterior communicating artery (PcomA) (49). Park et al. (63) and Yuan et al. (49) found that reducing coating length to ~1.5 mm reduced mortality, likely because the shorter tip does not interfere with PcomA blood flow (Figure 1B). Additionally, Amki et al. (64) proposed that damage in the thalamus, hypothalamus, and hippocampus (Figure 2F) was associated with occlusion of deeper and smaller arteries, such as the AchA, VTA, and LHA, which means that when another artery, besides the MCA is occluded, lesion volume and morphology are affected. Another problem associated with this model is high mortality levels after surgery, which can reach 83 %(19). Indeed, the majority of studies employing this model reported very discrepant mortality values (63, 65–68), but no correlation between occlusion time and mortality rate was established. Our results are in accordance with published data, since the group with longer occlusion time presented lower mortality rate and less variable lesions. The coating size of the filaments (1–2 mm) used for longer occlusions only affect the MCA territory. Moreover, another important advantage of our study is the use of commercial filaments, providing greater precision in the coating size of the filament. Furthermore, the use of commercial standardized filaments is critical to obtain consistent infarct volumes. “Home-made” filaments lead to inconsistent coating (both in length and thickness) according to previous experiments, resulting in more variability in the affected brain region. In our study, we used the histological TTC to evaluate lesion size; however, it is already possible to perform MRI (magnetic resonance imaging) on these preclinical models, which is an advantage, allowing for longitudinal studies with the same animal and detailed 3D brain lesion evaluation similar to patients with stroke. Thus, if available, it will be a better tool for translational studies.

The use of postoperative care protocols aims to maximize long-term survival and to mimic the care provided to patients with stroke (24). The protocol implemented here was optimized considering what was described by Modo et al. (14) and Ryan et al. (17) but with important modifications. The described procedures control temperature, hypoglycemia, and pain, and include manual/enteral feeding and nutrition and fluid supplementation (69), as well as social stress management (home cage and familiar cage mates). Patients with stroke suffer from weight loss associated with dysphagia, depression, aspiration, decreased dietary intake, and other eating difficulties, and weight loss has been associated with stroke severity, as measured by the NIHSS (70). Thus, implementing the described intensive postoperative care is a critical asset to improve this model, leading to faster recovery (as seen in weight evaluation) and contributing to lower mortality rate (6.6%).

Composite functional scores are used to assess general post-surgical impairments by allowing the monitoring of ischemic deficits, as well as assessment of recovery and therapy effectiveness (10, 14). The neurologic scale established for mice subjected to a stroke model can be compared to the mRS scale. According to what was previously described, we demonstrate that the tMCAO mice present neurological scores of ~16 (Figure 2D), showing that the composite score is reliable for evaluation of stroke effects (10, 48).

Several functional tests have been conducted in translational stroke research, but not all provide equal, efficient, and valid results (5, 71). In humans, stroke severity evaluation and prognosis may be accomplished using mRS (to measure chronic post-stroke neurological disability), NIHSS (to assess efficacy of acute stroke treatments), and other neurologic scales. The neurologic scale (modified DeSimoni) employed in this mouse model 2 h post-reperfusion evaluates parameters like gait, grooming, strength, and posture (11), making it comparable to clinical values obtained with the mRS scale. The mRS scale evaluates the post-stroke period (3–6 months) and is considered the most sensitive end point for clinical trials (7, 8), whereas NIHSS is used to determine the correct treatment in the acute phase and to assess the efficacy of stroke treatments in the first weeks.

In clinics, patients show decreased postural control, balance deficits, hemiparesis, and limb neuromuscular incoordination, compromising reaching, gait patterns, and walking speed, and increasing the risk of falling (54). The motor tests presented in this study (rotarod, pole, cylinder, ladder rung, and open field) evaluate similar parameters in mice. The rotarod, widely used in MCAO models (5, 72), evidenced motor coordination deficits in the tMCAO mice (Figure 3C). The pole test, which evaluates mice's motor coordination at early and late time points after stroke, clearly pointed deficits in the tMCAO group (Figure 3B). By performing the cylinder test, as Li et al. (41) demonstrated, a correlation between infarct volume and relatively severe injuries (1, 8, 72) was also observed, with the tMCAO mice showing a significantly reduced asymmetry score in forelimb use (Figure 3D). The ladder rung walking test provided a fine-adjusted muscle and limb coordination assessment (34, 35), demonstrating that the tMCAO animals have increased misplacements of the ipsilateral limbs (Figure 3E), as well as impairments in aiming movements and digit flexing.

Patients with stroke also present, during recovery, compromised levels of consciousness, dysphagia, depression, and sensory/perceptual deficits (54), which we were also able to evaluate in mice by conducting cognitive and sensorimotor tests (sunflower seed, adhesive removal, open field, and NOR). Rewell et al. (6) reported that tMCAO rodents struggle in breaking sunflower seeds into smaller pieces, leaving them uneaten, as we also observed in this study (Figure 4B). In the adhesive removal test, the tMCAO mice clearly showed an impairment in forelimb movement and coordination, which is associated to damage to the sensorimotor cortex or the striatum (Figure 4C) (5, 13, 32, 71). Moreover, the tMCAO animals show a sensory bias toward making contact and removing the adhesive with the contralateral side first, a clear ischemia-induced sensorimotor asymmetry that reflects failure to respond to ipsilateral tactile stimulus.

During the acute phase of focal ischemia, the mice develop a hypoactive phenotype in the open field arena and a turning preference to unaffected body side (5, 72, 73), as we reported in our model, on day 5 post-ischemia (Figure 5A). Hunter et al. (74) also described that permanent MCAO mice showed a significant reduction in locomotor activity.

We also found that the tMCAO and sham animals display anxiety-like behaviors in the open field test, a probable consequence of the surgery. However, the sham mice's motivation should be considered, and how they explore the open field vertically (rearing) seems to indicate that their “mental state”/motivation is higher than that of the tMCAO animals. In order to study stroke-caused depression, conducting other functional tests to specifically evaluate anxiety-like behaviors is advised, such as the elevated plus maze or the light-dark test (56).

We reported evident cognitive deficits, in the tMCAO mice (Figure 5C). However, the NOR test relies on mice's spontaneous exploratory behavior (13, 75), and because the tMCAO mice explore the objects for shorter periods than the control and sham mice, therefore, this test was difficult to establish, and there was a high exclusion rate of mice that did not accomplish the test goal (~35%). In order to overcome these issues, other behavioral tests to assess memory (e.g., passive avoidance or Barnes maze) can be performed.

**Figure 6:**
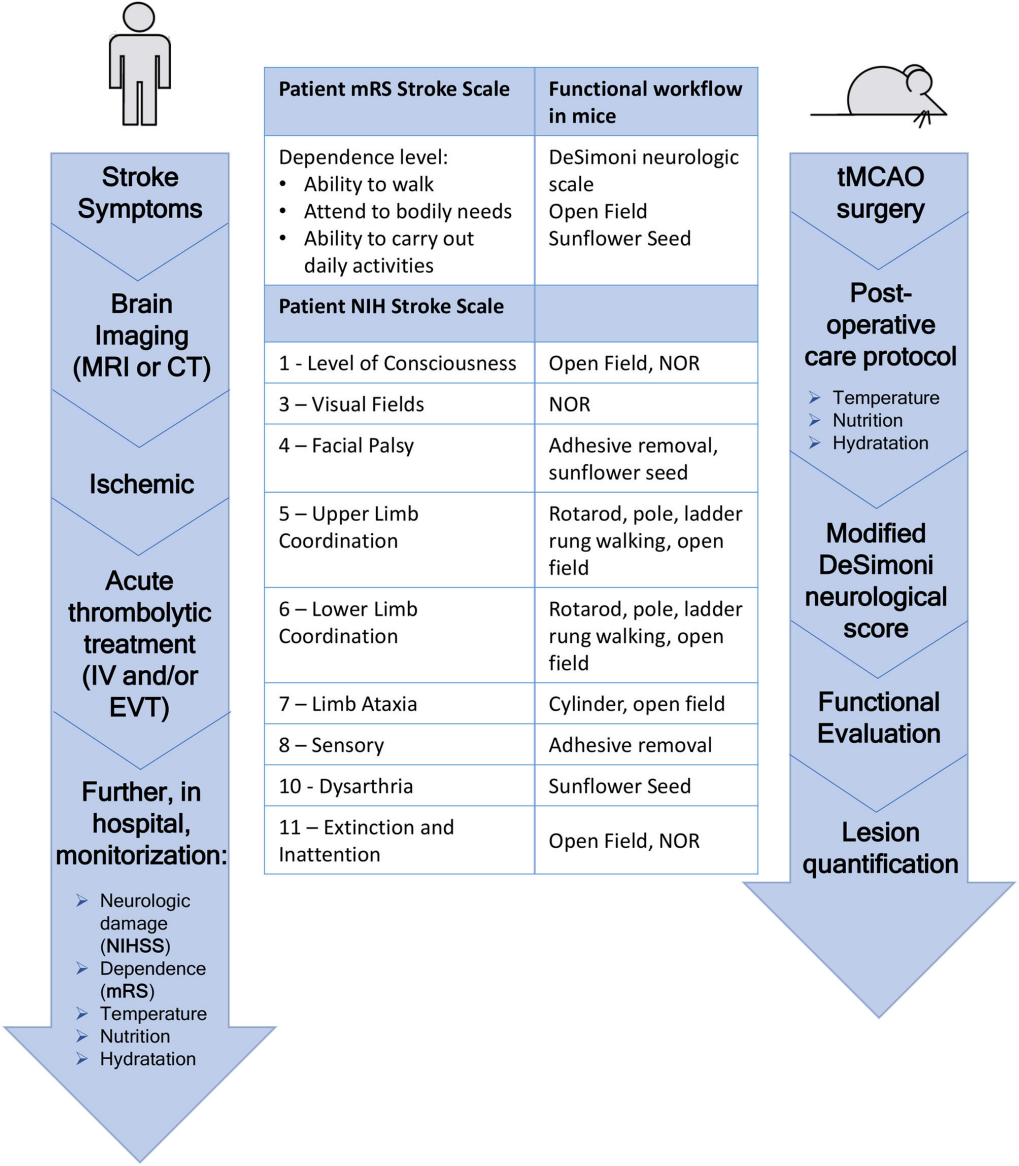
Flowchart of patient treatment of stroke symptoms compared to the flowchart of the timeline followed in this mouse stroke model. Direct correlation between several tasks of the patient's NIH stroke scale and the tests compromised in the behavior workflow can be seen.

In rodents, functional impairments after stroke can be divided into transient neurological deficits and long-term motor and cognitive dysfunctions. Although our behavioral planning only comprises a 7-day assessment of ischemic deficits, we believe that a potential therapeutic drug/strategy could be tested for 1 week as a first approach and then expanded to 30 days if successful. Some of the described tests lack sensitivity for a long-term workflow, such as the rotarod (71). Nonetheless, the cylinder test was shown to discriminate the sham mice from the tMCAO mice for up to 2 weeks, the pole test for up to 3 weeks, and the adhesive and ladder rung for up to a month post-ischemia (5). Also, fine motor coordination and cognition tests (seed, open field, and NOR) allow for the discrimination of stroke effects in months-long assessments on rodents. Thereby, the described battery of functional tests can be applied for month-long monitoring of tMCAO impairment/recovery in preclinical stroke studies, including other mouse stroke models that involve clear motor impairments (like permanent models). Importantly, the tests to assess sensorimotor function should be the primary end point to evaluate a treatment effect, since these are the more relevant functional impairments observed in patients with stroke (Figure 6).

Taken together, we described an improved intraluminal tMCA occlusion model in C57/BL6 mice, with reduced mortality and consistent infarct area, by controlling the internal arteries that are occluded and applying a multi-parameter intensive post-stroke care, taking clinical practice of patients with stroke as a reference. Additionally, we described and tested an innovative and comprehensive battery of functional tests to assess short-term sensorimotor and cognitive deficits related to ischemic stroke, which is parallel to the NIHSS and mRS scales used in patients with stroke (Figure 6). In conclusion, selection of appropriate occlusion filaments, post-stroke care similar to patients, and an implemented battery of relevant functional tests ranging from sensorimotor to cognitive functions, allow for the development of an optimized preclinical mouse stroke model, ideal to assess stroke outcomes and potential therapeutic strategies, and, importantly, with more successful translation to human clinical trials.

## Data Availability Statement

The original contributions presented in the study are included in the article/Supplementary Material, further inquiries can be directed to the corresponding author.

## Ethics Statement

All experiments and procedures were approved by the Institutional and National General Veterinary Board Ethical Committees (Approval Reference Number 003424), and are in line with National and European Union rules.

## Author Contributions

RP and JG made a substantial contribution in concept and design and in the acquisition and analysis/interpretation of the data. AM, LM, and MS contributed in the acquisition and analysis/interpretation of the data. PB and AL contributed in the analysis/interpretation of the data. All the authors drafted the article and revised and approved the version to be published.

## Funding

This study was supported by FEDER–COMPETE and National Funding from the Portuguese Foundation for Science and Technology (FCT) under the Projects PEST-c/SAU/LA0002/2011, FCT-FEDER for Unit 4293 (PT2020), UIDB/04293/2020, and Norte-01-0145-FEDER-000008.

## Conflict of Interest

The authors declare that the research was conducted in the absence of any commercial or financial relationships that could be construed as a potential conflict of interest.

## Publisher's Note

All claims expressed in this article are solely those of the authors and do not necessarily represent those of their affiliated organizations, or those of the publisher, the editors and the reviewers. Any product that may be evaluated in this article, or claim that may be made by its manufacturer, is not guaranteed or endorsed by the publisher.

## Abbreviations

tMCAO: transient middle cerebral artery occlusion
MCA: middle cerebral artery
EVT: endovascular thrombectomy
IVT: intravenous thrombolysis
ACA: anterior carotid artery
AChA: anterior choroidal artery
BA: basilar artery
CCA: central carotid artery
ECA: external carotid artery
ICA: internal carotid artery
HTA: hypothalamic artery
MCA: middle cerebral artery
PCA: posterior cerebral artery
PcomA: posterior communicating artery
SCA: superior cerebellar artery
VA: vertebral artery
VTA: ventral thalamic artery
WT: wild type.
