# Supplementary material for: Bridging the Transient Intraluminal Stroke Preclinical Model to Clinical Practice: From Improved Surgical Procedures to a Workflow of Functional Tests
Source: Front Neurol. 2022 Mar 11;13:846735. doi: 10.3389/fneur.2022.846735 (PMC8963503; doi:10.3389/fneur.2022.846735)
Supplement: Supplementary file 2 [file Data_Sheet_1.docx]

**Supplementary Material**

**Methods**

**Functional tests**

**-Wire Hanging Test**

The wire hanging test was performed as previously described in [3-7], to measure grasping ability and forelimb strength, with slight modifications. The test apparatus consisted of a steel wire (3 mm diameter) stretched between two posts 50 cm above a type III cage with the bottom filled with normal bedding. Four days after the surgery, animals were made to grasp the wire with their forepaws, and the latency to fall was recorded. The maximum test duration was fixed at 60 seconds. A score of zero was assigned to animals that fell immediately, and a score of 60 seconds was assigned to animals that did not fall. Hindlimbs were gently covered with sticky tape (8 x 8 mm in each limb), to prevent the animal from using the four paws. The test was repeated three times for each animal, on day 4 after the surgery (D+4), with 10 minutes rest between trials. The falling latencies from the 3 trials were averaged. Analysis was performed on the videos obtained, à posteriori, frame-by-frame and by a blinded experimenter.

**Supplemental Tables:**

**Supplemental Table 1:** Relationship between animal body weight and required filament tip diameter for successful induction of focal cerebral ischemia

| **Animal weight (g)** | **Filament tip diameter (mm)** |
| --- | --- |
| **21-23** | 0.21 |
| **23-25** | 0.22 |
| **25-35** | 0.23 |
| **36-45** | 0.24 |

**Supplemental Table 2:** Comparison of stroke patient’s in-hospital care to both post-operative (simple vs improved) protocols employed in mice during this study.

| **Stroke patients in hospital care** | **tMCAO mice model optimized post-operative care** | **tMCAO mice model simple post-operative care** |
| --- | --- | --- |
| Hypoglycaemia control | 2x/day syringe feeding with supplement AnimaStrath® [9] |  |
| Diet | Mashed pellets and nutritionally fortified water gel in cage floor Before/After surgery [9-11] | Mashed pellets and nutritionally fortified water gel in cage floor AFTER surgery |
| Fluid supplement | 2x/day 5% glucose in 0.9% NaCl subcutaneous injection [9-12] | 2x/day 5% glucose in 0.9% NaCl subcutaneous injection (3 days post-stroke) |
| Pain control | Buprenorphine administered 2x/day, first 3 days [10]^,^ [12]^,^ [9]^,^ [11] | Buprenorphine administered 2x/day, first 3 days |
| Supplemented diet | 2x/day 50% Duphalyte subcutaneous injection [9] | 2x/day 50% Duphalyte subcutaneous injection (3 days post-stroke) |
| Stress control | Pre-surgery cage mates were maintained and paper/rolls from the original cage [9]^,^ [12]^,^ [11] |  |
| Temperature monitorization | Recovering box:  35° C, 2h  33° C, 12h [13]^,^ [14]^,^ [11] | Recovering box:  35° C, ON  33° C, 24h |

**Supplemental Table 3.** Summary of the statistical analyses used in this study

| **Figure** | **Sample size** | **Statistical Methods** | **Degrees of freedom and F/t/p value** | **Post-hoc test** | **Significance** |
| --- | --- | --- | --- | --- | --- |
| 2C | Sham=6, tMCAO 25min 2-3mm=5, tMCAO 45min 1-2mm =6 | One-Way ANOVA | F (DFn, DFd): F (2, 14) = 43.46, P<0,0001 | Sidak’s multiple  comparison | Sham vs tMCAO 25min, p= <0,0001; Sham vs tMCAO 45min, p= <0,0001; tMCAO 25min vs tMCAO 45min, p= 0.7434 |
| 2D | Sham=3, tMCAO 25min 2-3mm=4, tMCAO 45min 1-2mm =5 | One-Way ANOVA | F (DFn, DFd): F (2, 9) = 16.74, P=0.0009 | Sidak’s multiple  comparison | Sham vs tMCAO 25min, p=0.0047; Sham vs tMCAO 45min, p=0.0010; tMCAO 25min vs tMCAO 45min, p= 0.7007 |
| 2F | Sham=10, Normal PO Care (NPC)=15, Optimized PO Care (OPC)=13 | Two-Way ANOVA | Day effect: F(7,232)= 74.63, df=2; p= <0.0001 | Tukey’s multiple comparation test | **Day 3**: Sham vs OPC, p=0.0327; **Day 4**: Sham vs OPC, p= <0,0001; OPC vs NPC, p=0.0305; **Day 5**: Sham vs OPC, p=0.0002; **Day 6:** Sham vs OPC, p=0.0003; OPC vs NPC, p=0.0019; **Day 7**: Sham vs OPC, p=0.0064, NPC vs OPC, p= <0,0001 |
| 3B | CT=4, Sham=14, tMCAO=14 | Two-Way ANOVA | Animal group effect: F(2,58)= 6.871, df=2; p= 0.0021 | Tukey’s multiple comparation test | **Time to turn****:** Ct vs Sham, p=0.9203; Ct vs tMCAO, p=0.5431; Sham vs tMCAO, p=0.0848  **Time to descend:** Ct vs Sham, p=0.8516; Ct vs tMCAO, p=0.3169; Sham vs tMCAO, p=0.0108 |
| 3C | CT=4, Sham=14, tMCAO=14 | One-Way ANOVA | F (2, 29) = 13.55, p= <0.0001 | Tukey’s multiple comparation test | Sham vs tMCAO, p= <0.0001; CT vs tMCAO, p=0.0159; Ct vs Sham, p=0,9181 |
| 3D | CT=12, Sham=17, tMCAO=17 | One-Way ANOVA | F (2, 43) = 12.83, p= <0.0001 | Tukey’s multiple comparation test | Sham vs tMCAO, p= <0.0001; CT vs tMCAO, p=0.0028; Ct vs Sham, p=0,6606 |
| 3E (Total Error) | CT=4, Sham=14, tMCAO=14 | One-Way ANOVA | F (2, 29) = 8.412, P=0.0013 | Tukey’s multiple comparation test | Sham vs tMCAO, p=0.0025; CT vs tMCAO, p=0.0191; Ct vs Sham, p=0,9069 |
| 3E (Digit score) | CT=4, Sham=14, tMCAO=14 | Two-Way ANOVA | Side effect: F(1,58)= 5.415, df=1; p= 0.0235  Condition effect: F(2,58)= 13.40, df=2; p=<0.0001 | Tukey’s multiple comparation test | Ipsilateral tMCAO vs Contralateral tMCAO, p=<0.0001; Ipsilateral CT vs Ipsilateral tMCAO, p=<0.0001; Ipsilateral Sham vs Ipsilateral tMCAO, p=<0.0001 |
| 3F (Total Distance) | CT=12, Sham=14, tMCAO=14 | One-Way ANOVA | F (2, 37) = 3.840, P=0.0305 | Tukey’s multiple comparation test | CT vs tMCAO, p=0.0260; Sham vs tMCAO, p=0.6724 |
| 3F (Resting Time) | CT=12, Sham=14, tMCAO=14 | One-Way ANOVA | F (2, 37) = 8.621, P=0.0008 | Tukey’s multiple comparation test | CT vs tMCAO, p=0.0009; Ct vs Sham, p=0.0096 |
| 3F (Mean Speed) | CT=12, Sham=14, tMCAO=14 | One-Way ANOVA | F (2, 37) = 4.990, P=0.0121 | Tukey’s multiple comparation test | CT vs tMCAO, p=0.0108; Ct vs Sham, P=0.0730; Sham vs tMCAO, p=0.6823 |
| 3F (R/L turn ratio) | CT=12, Sham=14, tMCAO=14 | One-Way ANOVA | F (2, 37) = 23.76, p=<0.0001 | Tukey’s multiple comparation test | CT vs tMCAO, p=<0.0001; Sham vs tMCAO, p=<0.0001; Ct vs Sham, p=0.9621 |
| 4B (Uneaten Seeds) | CT=4, Sham=14, tMCAO=14 | One-Way ANOVA | F (2, 29) = 20.71, p=<0.0001 | Tukey’s multiple comparation test | CT vs tMCAO, p=0.0005; Sham vs tMCAO, p=<0.0001 |
| 4B (Shell pieces) | CT=4, Sham=14, tMCAO=14 | One-Way ANOVA | F (2, 29) = 5.272, P=0.0248 | Tukey’s multiple comparation test | CT vs tMCAO, p=0.0041; Sham vs tMCAO, p=0.028; Ct vs Sham, p=0.5396 |
| 4C (Time to contact) | CT=4, Sham=14, tMCAO=13 | Two-Way ANOVA | Side effect: F(1,56)= 1.751, df=1; p= 0.1911  Condition effect: F(2,56)= 15.13, df=2; p=<0.0001 | Tukey’s multiple comparation test | Ipsilateral tMCAO vs Contralateral tMCAO, p=0.0003; Ipsilateral CT vs Ipsilateral tMCAO, p=0.0014; Ipsilateral Sham vs Ipsilateral tMCAO, p=<0.0001 |
| 4C (Time to remove) | CT=4, Sham=14, tMCAO=13 | Two-Way ANOVA | Side effect: F(1,56)= 3.958, df=1; p=0.0515  Condition effect: F(2,56)= 16.14, df=2; p=<0.0001 | Tukey’s multiple comparation test | Ipsilateral tMCAO vs Contralateral tMCAO, p=<0.0001; Ipsilateral CT vs Ipsilateral tMCAO, p=0.0004; Ipsilateral Sham vs Ipsilateral tMCAO, p=<0.0001 |
| 5B (Distance) | CT=12, Sham=14, tMCAO=14 | Two-Way ANOVA | Condition effect: F(2,74)=0.4145, df=2; p=0.6622  Zone effect: F(1,74)= 786.0, df=1; p=<0.0001 | Tukey’s multiple comparation test | **Center:** Ct vs Sham. P=0.2812; CT vs tMCAO, p=0.1906  **Pheriphery:** Ct vs Sham. P=0.8952; CT vs tMCAO, p=0.1906 |
| 5B (Rearings) | CT=12, Sham=14, tMCAO=14 | One-Way ANOVA | F (2, 37) = 1.60, P=0.0001 | Tukey’s multiple comparation test | CT vs tMCAO, p=0.0001; Sham vs tMCAO, p=0.0043 |
| 5B (Grooming) | CT=12, Sham=14, tMCAO=14 | One-Way ANOVA | F (2, 37) = 5.537, P=0.0079 | Tukey’s multiple comparation test | CT vs tMCAO, p=0.0074; CT vs Sham, p=0.05; Sham vs tMCAO, p=0.6994 |
| 5C | CT=8, Sham=17, tMCAO=13 | One-Way ANOVA | F (2, 35) = 7.950, P=0.0014 | Tukey’s multiple comparation test | CT vs tMCAO, p=0.0028; Sham vs tMCAO, p=0.0081; Ct vs Sham, p=0.5664 |
| Suppl. Fig 2A | CT=4, Sham=14, tMCAO=14 | Two-Way ANOVA | Ipsilateral:  Step effect: F(6,203)= 116.2, df=6; p=<0.0001  Condition effect: F(2,203)= 0.00036, df=2; p=0.9996  Contralateral:  Step effect: F(6,203)= 96.61, df=6; p=<0.0001  Condition effect: F(2,203)= 2.158e-014, df=2; p=0.9999 | Sidak’s multiple comparation test |  |
| Correct Placement |  |  |  |  | **Ipsilateral:**  CT vs tMCAO, p=<0.0001; Sham vs tMCAO, p=<0.0001  **Contralateral:**  CT vs tMCAO, p=0.0005; Sham vs tMCAO, p=0.0024 |
| Partial Placement |  |  |  |  | **Ipsilateral:**  CT vs tMCAO, p=0.0064; Sham vs tMCAO, p=0.0168  **Contralateral:**  Sham vs tMCAO, p=0.0005 |
| Slight slip |  |  |  |  | **Ipsilateral:**  CT vs tMCAO, p=0.0023; Sham vs tMCAO, p=<0.0001 |
| Suppl. Fig 2B | CT=4, Sham=14, tMCAO=14 | Two-Way ANOVA | Limb effect: F(3,116)= 1.907, df=3; p= 0.1323  Condition effect: F(2,116)= 5.694, df=2; p=0.0044 | Tukey’s multiple comparation test | **Ipsilateral forelimb:**  CT vs tMCAO, p=0.0043; Sham vs tMCAO, p=<0.0001  **Ipsilateral hindlimb:**  CT vs tMCAO, p=0.7080; Sham vs tMCAO, p=0.3929 |

**Graphical abstract – Improvement of the surgical, post-operative and functional evaluation in the tMCAO mice give rise to a highly paralleling and translatable into clinic model.** Induction of ischemia for 45-minutes using smaller filaments gives rise to uniform lesion volume that together with the improved post-operative care, decreases this model mortality. Furthermore, this allows for intensive long-term functional evaluation of mice. This way, clinical symptoms and ethology, as well as scales used to evaluate stroke, are mimicked in a mouse model.

**Supplementary figure 1- Preliminary studies let us conclude that occlusion of the MCA for smaller periods with smaller filaments does not give rise to infarct. In comparison, occlusion for longer periods, using the larger filaments leads to infarcts that occupy areas outside the MCA, such as the hippocampus.** Representative photography of TTC stained brain slices from tMCAO animals whose occlusion parameters were: 25-minute occlusion with 1-2mm filament (left panel) and 45-minute occlusion with 2-3mm filament (right panel);

**Supplementary figure 2 – The ladder rung shows fine movement deficits in the seven stepping categories scale in tMCAO animals, while sham does not differ from WT. (A)** The ladder rung allows to evaluate each step each mouse performs with each discriminated paw; **(B)** This test also allows to evaluate mice’s misplacement of each individual paw (error frequency for each paw as a percent of total steps); WT n=4 (4 females), sham n=14 (4 males and 10 females), tMCAO n=14 (9 males and 5 females). ****p<0.0001, ***p<0.001, **p<0.01, *p<0.05. Two-way ANOVA followed by Tukey’s multiple comparison test.

**Supplementary figure 3 – The Wire Hanging test does not differentiate tMCAO animals from controls.** Time, in seconds that took the animals to fall from the wire. WT n=4 (4 females), sham n=3 (1 males and 2 females), tMCAO n=5 (4 males and 1 female). ****p<0.0001, ***p<0.001, **p<0.01, *p<0.05. One-way ANOVA followed by Tukey’s multiple comparison test.

**Supplementary video 1 – Video comparing the performance of tMCAO, sham and WT animals in the pole test.** tMCAO animals take much longer to turn and descend the pole when compared to the controls.

**Supplementary video 2 – Video showing the cylinder recording angle.** The cylinder test was recorded from above, ensuring that all the walls were seen and that there was high contrast between animal and background.

**Supplementary video 3 – Video comparing the performance of tMCAO, sham and WT animals in the ladder rung walking test.** tMCAO animals take longer to cross the ladder due to the slips and errors in stepping parameters while crossing the ladder.

**Supplementary video 4 -** **Video comparing the performance of tMCAO, sham and WT animals in the adhesive removal test.** tMCAO animals take much longer to contact and remove the adhesive from the ipsilateral paw compared to the controls.

Bibliography

[3] Hattori K, Lee H, Hurn PD, Crain BJ, Traystman RJ, DeVries AC. Cognitive deficits after focal cerebral ischemia in mice. Stroke. 2000;31:1939-44.

[4] Gertz K, Priller J, Kronenberg G, Fink KB, Winter B, Schrock H, et al. Physical activity improves long-term stroke outcome via endothelial nitric oxide synthase-dependent augmentation of neovascularization and cerebral blood flow. Circ Res. 2006;99:1132-40.

[5] Prinz V, Laufs U, Gertz K, Kronenberg G, Balkaya M, Leithner C, et al. Intravenous rosuvastatin for acute stroke treatment: an animal study. Stroke. 2008;39:433-8.

[6] Royl G, Balkaya M, Lehmann S, Lehnardt S, Stohlmann K, Lindauer U, et al. Effects of the PDE5-inhibitor vardenafil in a mouse stroke model. Brain Res. 2009;1265:148-57.

[7] De Silva TM, Brait VH, Drummond GR, Sobey CG, Miller AA. Nox2 oxidase activity accounts for the oxidative stress and vasomotor dysfunction in mouse cerebral arteries following ischemic stroke. PLoS One. 2011;6:e28393.

[9] Ryan CL, Doucette TA, Gill DA, Langdon KD, Liu Y, Perry MA, et al. An improved post-operative care protocol allows detection of long-term functional deficits following MCAo surgery in rats. Journal of Neuroscience Methods. 2006;154:30-7.

[10] Mhairi Macrae I. Focal ischemia models: Middle cerebral artery occlusion induced by electrocoagulation, occluding devices, and endothelin-1. 2016. p. 45-58.

[11] Percie du Sert N, Alfieri A, Allan SM, Carswell HV, Deuchar GA, Farr TD, et al. The IMPROVE Guidelines (Ischaemia Models: Procedural Refinements Of in Vivo Experiments). J Cereb Blood Flow Metab. 2017;37:3488-517.

[12] Modo M, Stroemer RP, Tang E, Veizovic T, Sowniski P, Hodges H. Neurological sequelae and long-term behavioural assessment of rats with transient middle cerebral artery occlusion. Journal of Neuroscience Methods. 2000;104:99-109.

[13] Wu L, Xu L, Xu X, Fan X, Xie Y, Yang L, et al. Keep warm and get success: the role of postischemic temperature in the mouse middle cerebral artery occlusion model. Brain Res Bull. 2014;101:12-7.

[14] Barber PA, Hoyte L, Colbourne F, Buchan AM. Temperature-regulated model of focal ischemia in the mouse: a study with histopathological and behavioral outcomes. Stroke. 2004;35:1720-5.
