# Supplementary figures and images for: Bridging the Transient Intraluminal Stroke Preclinical Model to Clinical Practice: From Improved Surgical Procedures to a Workflow of Functional Tests
Source: Front Neurol. 2022 Mar 11;13:846735. doi: 10.3389/fneur.2022.846735 (PMC8963503; doi:10.3389/fneur.2022.846735)

Supplemental Fig. 1

A

tMCAO 25-min  
1-2mm

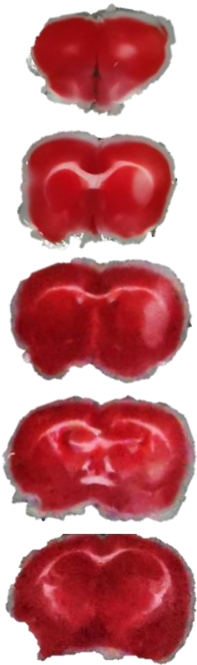

B

tMCAO 45-min  
2-3mm

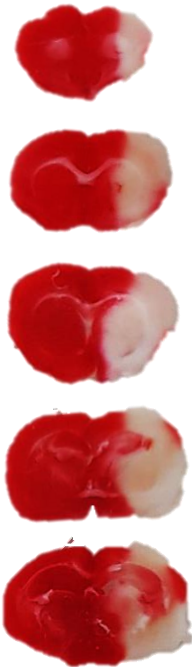

**Supplemental Fig. 2**

**A**

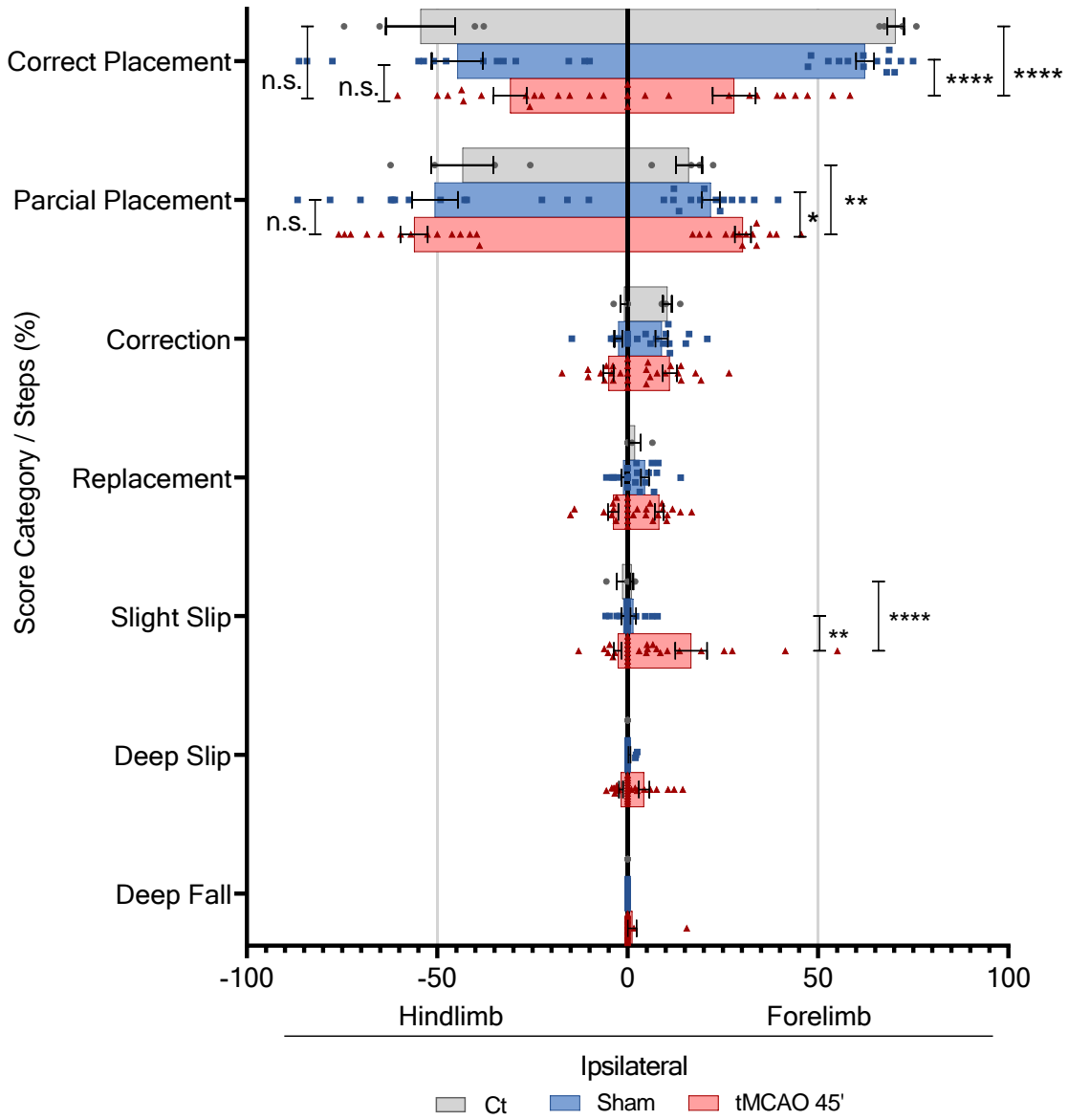

**B**

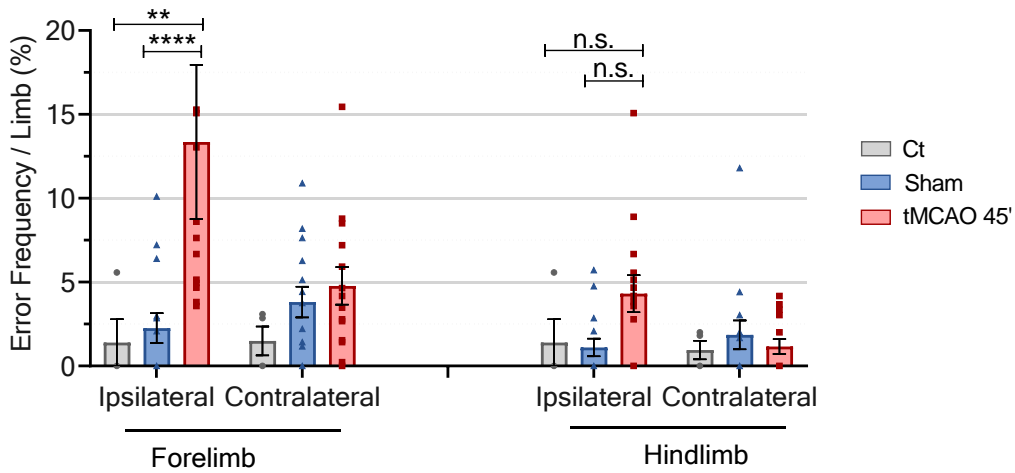

**Supplemental Fig. 3**

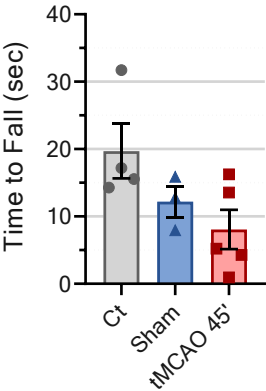

Supplement: Supplementary file 3 [file Image_1.pdf]
